# Alkaline Earth Metal Fluorides (MgF_2_, CaF_2_, SrF_2_, BaF_2_) and Nb_2_O_5_ Effect on the Structural and Optical Properties of New Fluorophosphoniobate Glasses

**DOI:** 10.1021/acsomega.5c05892

**Published:** 2025-09-26

**Authors:** Leandro Olivetti Estevam da Silva, Lais Dantas Silva, Edgar Dutra Zanotto, Marcos de Oliveira Junior, Danilo Manzani

**Affiliations:** † São Carlos Institute of Chemistry (IQSC), 117186 University of São Paulo (USP), São Carlos, SP 13566-590, Brazil; ‡ Department of Chemistry and Biology, State University of Maranhão (UEMA), Caxias, MA 65604-380, Brazil; § Center for Research, Technology, and Education in Vitreous Materials (CeRTEV), Department of Materials Engineering (DEMa), 67828 Federal University of São Carlos (UFSCar), São Carlos, SP 13565-905, Brazil; ∥ São Carlos Institute of Physics (IFSC), University of São Paulo (USP), São Carlos, SP 13566-590, Brazil

## Abstract

Niobium-fluorophosphate glasses show promising technological and scientific potential in a wide range of optical and photonic applications due to their properties as a host matrix, such as wide transparency between the ultraviolet and near-infrared, high solubility to rare-earth ions, low phonon energy, and high chemical stability. Efforts were previously made to study the effects that different concentrations of niobium oxide have on the base phosphate glass composition used here in the structural, thermal, and optical properties. However, an exploration of which changes different alkaline earth metals can induce in niobium-phosphate glass properties, considering their modifying role and periodic properties, is lacking. Therefore, this study aimed to thoroughly investigate how different alkaline earth metals can induce variations in the structural, thermal, and optical properties of a novel niobium-phosphate glass. The tested glasses followed the compositional rule (80 – *y*)­NaPO_3_–*y*Nb_2_O_5_–20XF_2_ (X = Mg^2+^, Ca^2+^, Sr^2+^, Ba^2+^, *y* = 5, 10, 15, 20 mol % of Nb_2_O_5_) and were synthesized by the melt-quenching method. Analysis by differential scanning calorimetry (DSC), UV–vis absorption spectroscopy, and optical bandgap calculations shows that the covalent character of the glass matrix increases for increasing Nb_2_O_5_ content, causing an increase in the glass transition temperature, *T*_g_, and a decrease of the optical bandgap energy. DSC analyses revealed a very high stability against crystallization, Δ*T* up to nearly 400 °C (Δ*T* = *T*_x_ – *T*_g_)—where *T*_x_ is the crystallization peak temperature—for this glass-forming system. ^31^P NMR experiments revealed that the increase in Nb_2_O_5_ between 5 and 15 mol % induced the formation of P^0^, P_1Nb_^1^, and P_2Nb_^2^ phosphate units, consequently increasing the glass matrix connectivity due to the formation of P–O–Nb and Nb–O–Nb bonds. Moreover, ^19^F nuclear magnetic resonance showed how the alkaline earth metals with a higher charge-to-radius ratio (smaller ionic radius) preferentially bond with the fluoride species within the glass matrix. Consequently, the glass connectivity increases due to the lower availability of fluoride to interact with the main glass former groups (i.e., phosphate and niobate groups).

## Graphical abstract

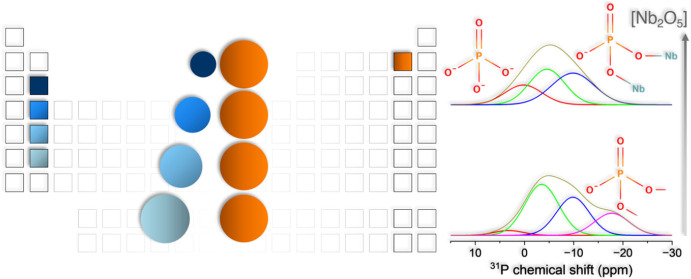

## 1 Introduction

Phosphate glasses are widely recognized for their properties of a broad transparency window spanning from UV to near-infrared, relatively low characteristic temperatures, and high solubility for rare-earth ions (RE^3+^) when compared to silicate glasses.^1–3^ Through chemical and structural modifications, it is always possible to achieve even better properties. The addition of fluoride precursors to form fluorophosphate glasses reduces the overall phonon energy and increases RE^3+^ solubility while preserving certain key features of oxide glasses, such as chemical stability.^4^ These properties make fluorophosphate glass matrices excellent candidates for applications in photonics.^5,6^ Due to the high volatility of fluoride compounds during the melting step at high temperatures, previous studies have focused on how synthesis conditions—such as melting temperature, time, and crucible material—influence the amount of fluorine lost and its preferential coordination with other species.^7^ In fluorophosphate glasses, fluoride induces significant alterations in the bonding environment, depending on the F/O ratio, by determining the distribution and connectivity of Q*^n^* phosphate groups throughout the glass structure.^8^ Therefore, fluorination strategies for phosphate-based glass compositions must be thoroughly controlled to obtain the desired properties in the resulting fluorophosphate glass. These properties include improved transparency in the UV range and a reduced mean phonon energy of the matrix, which is highly beneficial for minimizing nonradiative decay in luminescent rare-earth-doped matrices used in photonics, such as the composition studied here.^7–11^ Previous studies have demonstrated that incorporating certain metal oxides, such as TiO_2_, Nb_2_O_5_, and WO_3_, enhances the linear and nonlinear optical properties of phosphate glasses.^12–14^ Also, adding different concentrations of Nb_2_O_5_ to lead pyrophosphate-based glasses significantly increases the glass transition temperature as well as the linear and nonlinear refractive indices of the resulting glass.^13^ The content of [NbO_6_] units formed within the phosphate chains is highly dependent on the Nb_2_O_5_ content, which initially increases thermal stability against crystallization before decreasing it due to clustering.^13^ Furthermore, higher concentrations of Nb_2_O_5_ in phosphate glass compositions reduce hygroscopicity and enhance chemical stability.^12^ These effects demonstrate promising features for the optical applications of the resulting glass, combining the advantages of fluorophosphate matrices with the Nb_2_O_5_ dual role as a glass former and modifier.^4,6^ Additionally, Brazil holds very large reserves of Nb, making it important to gain a deep understanding of this metal in glasses and explore new products and technologies for the national development.^15^

Alkali and alkaline earth metals modify the phosphate glass network in different manners by producing or stabilizing nonbridging oxygens (NBO) and lone electron pairs, thereby changing the network connectivity through electrostatic forces.^16^ In-depth investigations have shown that different alkali and alkaline earth metals can significantly impact the structural, thermal, optical, and spectroscopic properties of RE^3+^-doped glasses in different proportions, following a periodic trend along the group, such as in the ionic radii, electron affinity, ionization energy, and polarizability.^17,18^ Although several studies have highlighted the impact of alkaline earth metals on glass properties, this work aims to have a comprehensive understanding regarding how their periodic properties change the properties and features of the resulting glasses.^19–21^ In this sense, this systematic study focuses on the investigation of both the effects by varying the Nb_2_O_5_ concentration and the type of alkaline earth metal added into a sodium metaphosphate-based glass. The most important contribution is to provide knowledge of the periodic trends and their influence on the structural, optical, and thermal properties of niobium-fluorophosphate glasses.

## 2 Experimental Part

### 2.1 Synthesis and Characterizations of the Niobium-Fluorophosphate Glasses

Niobium-fluorophosphate glasses were synthesized by the conventional melt-quenching method according to the molar compositional rule (80 – *y*)­NaPO_3_–*y*Nb_2_O_5_–20XF_2_, with X = Mg, Ca, Sr, Ba, and *y* = 5, 10, 15, 20 mol %, as detailed in Table 1. The glass samples were labeled as Nb*y*-X. The chemicals sodium metaphosphate NaPO_3_ (Aldrich, 65–70% P_2_O_5_ basis), magnesium fluoride MgF_2_ (Aldrich, 99.9%), calcium fluoride CaF_2_ (Aldrich, 99.9%), strontium fluoride SrF_2_ (Aldrich, 99.9%), barium fluoride BaF_2_ (Aldrich, 99.9%), and niobium oxide Nb_2_O_5_ (CBMM, optical grade) were thoroughly weighed, mixed, and homogenized in an agate mortar. Each powder mixture was melted in a covered Pt/Au (95/5 mol %) crucible at 1050 °C for 30 min. The melt was poured into a preheated stainless steel mold at 250–350 °C (depending on the composition) and annealed for 4 h to relieve internal stress before slowly cooling to room temperature. After annealing, the glass samples were cut, some pieces were polished for optical measurements, and other pieces were ground into powder.

**Table 1:** Nominal glass composition, labels, and P/Nb ratio (X = Mg, Ca, Sr, Ba)

	Molar composition (mol %)			
Sample label	NaPO_3_	Nb_2_O_5_	XF_2_	P/Nb ratio
Nb5-X	75	5	20	7.5
Nb10-X	70	10		3.5
Nb15-X	65	15		2.2
Nb20-X	60	20		1.5

The optical characterization was carried out using UV–vis–NIR spectroscopy with a Shimadzu UV-3600 spectrophotometer, scanning between 200 and 1000 nm. From the UV–vis–NIR absorption spectra, the molar absorptivity coefficients were calculated. Plots of (α*h*ν)^1/2^, (α*h*ν)^2^, and ln­(α) as a function of the energy *h*ν (in eV) were used to determine the indirect and direct bandgap energy and Urbach’s energy of each sample. The thermal analysis was performed by differential scanning calorimetry (DSC) to determine the characteristic temperatures. The analysis was performed using a Netzsch STA F3 Jupiter instrument. Powdered glass samples (<20 μm) of each composition were heated from 200 to 1000 °C in platinum crucibles under an N_2_ atmosphere at a heating rate of 10 °C min^–1^. The resulting curves were analyzed using Proteus software.

For structural characterization, Raman spectroscopy was carried out using a Jobin-Yvon Horiba HR800 instrument operating with a He/Ne laser at 632.8 nm. A detailed solid-state nuclear magnetic resonance (NMR) analysis was conducted to elucidate the effect of the cation size of four alkaline earth fluorides on the glass structure. ^19^F magic-angle spinning (MAS) NMR spectra were recorded in an Agilent DD2 spectrometer operating at 5.64 T (corresponding to ^1^H Larmor frequency of 240 MHz), using 1.6 mm rotors spinning at 35 kHz with a DEPTH pulse sequence for background suppression,^22^ a 90° pulse length of 2.4 μs, relaxation delays of 120 s, and up to 128 scans. ^19^F chemical shifts are reported relative to CFCl_3_ using solid AlF_3_ as a secondary reference (−172 ppm). Solid-state ^31^P, ^23^Na, ^23^Na­{^31^P} REDOR, ^31^P­{^23^Na} REAPDOR, and ^31^P­{^93^Nb} RESPDOR NMR experiments were conducted on a Bruker Avance Neo spectrometer operating at 14.1 T (corresponding to a ^1^H Larmor frequency of 600 MHz), using a 2.5 mm Bruker probe spinning at 15 kHz. The ^31^P MAS spectra were acquired by using single-pulse excitation with a 90° pulse of 2.4 μs. A recycling delay of 300 s was used, which ensures complete recovery of the equilibrium magnetization. Up to 128 scans were accumulated for noise averaging. In a separate set of measurements, double-quantum filtered spectra were obtained using the 1-D refocused-INADEQUATE method.^23^ This experiment results in the selective detection of only those ^31^P nuclei that are involved in a P–O–P linkage (P^1^ and P^2^ units) and, therefore, gives rise to the excitation of double-quantum coherence through indirect ^31^P–^31^P spin–spin coupling. In contrast, the signals of isolated P^0^ units are suppressed by the appropriate receiver phase cycling. Experimental conditions were: spinning speed of 15 kHz, π/2 pulse length of 2.4 μs and relaxation delay of 100 s. The mixing time for DQ coherence creation was 16.6 ms, corresponding to a value of the indirect coupling constant ^2^ *J*(^31^P–^31^P) of 30 Hz. The ^31^P chemical shifts are reported relative to a BPO_4_ secondary reference (−29.3 ppm against an 85% H_3_PO_4_ aqueous solution). The ^23^Na MAS spectra were obtained using single-pulse excitation with a 90° pulse of 4.57 μs, with a recycle delay of 0.5 s and accumulating up to 16 scans. ^23^Na­{^31^P} rotational-echo double-resonance (REDOR)^24^ measurements were acquired by using π recoupling pulses on the ^31^P channel (pulse length of 4.572 μs) while obtaining rotor-synchronized ^23^Na spin echoes, with a 90° pulse length of 2.286 μs, with a recycle delay of 0.5 s.

^31^P­{^23^Na} rotational-echo adiabatic passage double-resonance (REAPDOR)^25^ experiments were carried out using a typical value for ^31^P π-pulse duration of 4.9 μs, a spinning frequency of 20.0 kHz, and a recycle delay of 100 s. Dipolar recoupling was achieved by ^23^Na pulses applied at a nutation frequency of 96 kHz (measured for solid NaF_3_) and for a duration of one-third of the rotor period (16.67 μs). ^31^P­{^93^Nb} dipolar recoupling experiments were performed using the wideband uniform rate smooth truncation–resonance rotational-echo saturation-pulse double-resonance (WURST-RESPDOR) pulse sequence,^26^ where saturation of the quadrupolar spin (in the nonobserved ^93^Nb channel) is accomplished by a frequency-swept WURST pulse.^27^ The WURST saturation pulse parameters were optimized through SIMPSON^28^ simulations, as reported in ref 29, and were fixed as follows: 8 rotor cycles duration (400 μs), shape parameter *N* = 80, sweep width of 450 kHz, and nutation frequency of 53.0 kHz.

^25^Mg MAS spectra were acquired in a 3.2 mm probe using a rotor-assisted population transfer (RAPT)^30^ approach for signal enhancement, using a rotor-synchronized Hahn-echo scheme for signal detection. Signal enhancement was provided by a wideband, uniform rate, smooth truncation (WURST) pulse^27^ for ST → CT (satellite to central transition) population transfer, applied prior to the Hahn-echo block.^31–33^ The parameters used for the acquisition of ^25^Mg in the present work were all optimized experimentally on an isotopically enriched CaMgSi_2_O_6_ glass sample. Magic angle spinning (MAS) was fixed at 20 kHz; for the ST inversion, we used a WURST-80 pulse with 1.0 ms of duration, 20 kHz sweep width, pulse-power corresponding to a nutation frequency of 12.5 kHz (as measured for solid MgO), and a frequency offset of 350 kHz. For the detection step, we have used π/2 and π pulses of 6.2 and 12.4 μs and an interpulse delay of 50 μs. ^25^Mg chemical shifts were referenced against aqueous MgCl_2_, using solid MgO as a secondary reference (δ = 26 ppm).

## 3 Results and Discussion

The glass samples were successfully obtained by using the melt-quenching method, exhibiting transparency in the visible range and absence of bubbles and fractures, as shown in Figure S1. The susceptibility of the 5 mol % Nb_2_O_5_ samples to atmospheric moisture varies with the alkaline earth metal. Weeks after synthesis, the Nb5–Ba sample underwent complete surface corrosion, resulting in total opacity. The Nb5–Sr and Nb5–Ca samples, however, showed only an initial onset of haziness (Figure S1), whereas the Nb5–Mg sample displayed no observable alteration. This trend was further confirmed after prolonged exposure to the environment (Figure S2), where only the Nb5–Mg sample maintained its transparency, with the others showing a progressive increase in surface alteration.

Recent studies, such as the one conducted by Sreenivasan et al.,^34^ demonstrate that in quaternary sodium–magnesium aluminosilicate glass systems, the progressive increase in MgO content induces significant alterations in the glass network and promotes liquid phase separation. Using nuclear magnetic resonance of ^29^Si and ^27^Al, the authors observed that the substitution of Na^+^ by Mg^2+^ leads to the conversion of Q^3^ silicon species into Q^2^, thus reducing the polymerization of the network. Additionally, they found that due to its higher field strength, Mg^2+^ acts not only as a network modifier but also as a charge compensator, causing a redistribution of Si^4^(mAl) species: there was a decrease in Q^4^ (4Al) and Q^4^ (3Al), units, along with a concomitant increase in the less coordinated Q^4^ (2Al) and Q^4^ (1Al) species. This structural reorganization favors the formation of silica- and alumina-rich domains, characterizing phase separation. These results reinforce the hypothesis that network-modifying cations with a higher electrostatic field strength, such as Mg^2+^, play a decisive role in inducing and amplifying phase separation in complex systems.

Petrovskii et al.^35^ observed that, in the Na_2_O–K_2_O–Nb_2_O_5_–SiO_2_ system, metastable liquid phase separation occurs mainly in glasses containing 15 mol % or more Nb_2_O_5_, resulting in the formation of a micro inhomogeneous structure. In this structure, there are regions enriched in Nb_2_O_5_ and alkali, which, upon further heating, crystallize to form NaNbO_3_ microcrystals.

Comparing the Nb*y*-Ba samples, higher Nb_2_O_5_ content reduces its hygroscopicity since it acts as a glass former above certain concentrations in phosphate-based matrices.^13,36^ The presence of different alkaline earth fluorides also significantly affects the chemical stability of the obtained glasses. The cationic potential of alkaline earth metals, e.g., the ionic charge over ionic radii, was calculated for Mg^2+^, Ca^2+^, Sr^2+^, and Ba^2+^, being 2.78, 2.00, 1.72, and 1.47 Å^–1^, respectively.^37^ Elements with higher cationic potential (ionic charge/radii ratio), such as Mg^2+^, stabilize the structure against water absorption, probably due to less depolymerization of the phosphate backbone chain. In contrast, alkaline earths with lower cationic field strength, such as Sr^2+^ and, especially, Ba^2+^, increase the phosphate-based network depolymerization and consequently increase their hygroscopicity.

Figure S3 presents the DSC thermograms obtained for all samples. The characteristic temperatures including the *T*_g_, *T*_*x*_, and *T*_p_—glass transition temperature (*T*_g_) measured at the inflection point of the endothermic peak (obtained from the first derivative of the DSC thermogram), onset crystallization temperature (*T*_x_), and maximum crystallization temperature (*T*_p_) obtained from the onset and peak of the exothermic event in the thermogram, respectively—were determined, and a parameter of glass stability against crystallization, Δ*T*, was obtained for each sample, and is listed in Table S1. Figure 1a shows the behavior of *T*_g_ as a function of the Nb_2_O_5_ content. The increased levels of Nb_2_O_5_ lead to increased *T*_g_ values, probably due to the formation of Nb–O–P and Nb–O–Nb linkages by the insertion of NbO_6_ units within the phosphate backbone chain, which are stronger than the bonds of the original glass former (P–O–P).^13,38^ Cicconi et al.^39^ demonstrated that the incorporation of Nb promotes the association of NbO_6_ units, initiating the formation of a subnetwork composed of vertex-sharing NbO_6_ octahedra. Similarly, Koudelka et al.^40^ state that, for low Nb_2_O_5_ contents, niobium atoms form isolated NbO_6_ octahedra. However, as the Nb_2_O_5_ concentration increases, these octahedra tend to cluster, leading to greater connectivity of the glass structure. It can be clearly seen in the Raman spectra analysis. On the other hand, when the size of the alkaline earth metal increases from Mg^2+^ to Ba^2+^, maintaining the Nb_2_O_5_ content, the *T*_g_ values decrease by increasing their ionic radii (*r*_Ba_^2+^ > *r*_Sr_ > *r*_Ca_^2+^ > *r*_Mg_). Hence, it appears that smaller ionic radii, such as those of Mg^2+^, promote a higher connectivity of the glass network and lower depolymerization compared with larger cations, thereby increasing chemical durability. The influence of alkaline earth metals’ ionic radius is depicted in Figure S4, which shows the values of *T*_g_ as a function of their radius and Nb_2_O_5_ content.

**Figure 1:**
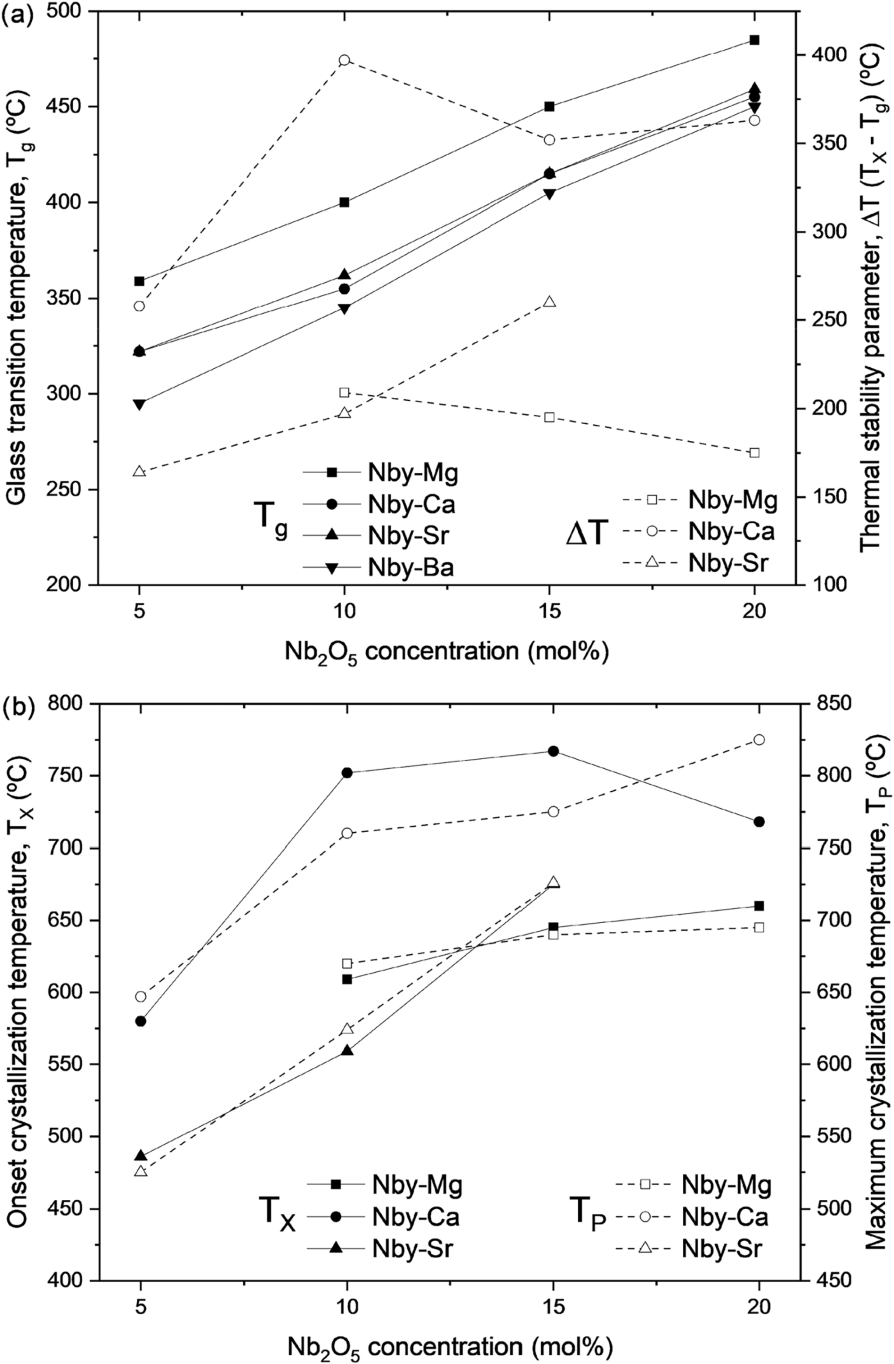
Behavior of (a) the glass transition temperature (*T*_g_, solid lines) and thermal stability against devitrification (Δ*T*, dashed lines), and (b) the onset crystallization temperature (*T*_x_, solid lines) and the maximum crystallization temperature (*T*_p_, dashed lines) for all Nb*y*-X samples. The temperatures were determined from DSC measurements.

Figure 1a depicts thermal stability against crystallization as a function of the alkaline earth and Nb_2_O_5_ content. Previous studies on the Pb_2_P_2_O_7_–Nb_2_O_5_ system revealed that *T*_g_ increases when the P/Nb ratio decreases, while Δ*T* increases up to P/Nb = 2 and then decreases with lower P/Nb ratios due to the formation of NbO*_n_* clusters. This was confirmed by Nb–O–Nb linkages that appeared in Raman spectra.^13,41^ Mošner et al. demonstrated that *T*_g_ and *T*_x_ increase when P_2_O_5_ is replaced by Nb_2_O_5_ in the Na_2_O–P_2_O_5_–Nb_2_O_5_ binary glass system, while the Δ*T* parameter decreases.^36,42^ Stunda-Zujeva et al. studied the effect of adding CaO to the Na_2_O–P_2_O_5_–Nb_2_O_5_ system. The decrease in the P/Nb ratio increased the Δ*T* values up to 125 °C and subsequently decreased them down to 92 °C.^43^ Similar results were obtained for the Nb–Ca samples in this study. Increasing the Nb_2_O_5_ content from 5 to 10 mol % led to a significant increase in the Δ*T* values, from 258 to 397 °C. However, a further increase in Nb_2_O_5_ to 15 and 20 mol % resulted in a decrease of the Δ*T* values, which reached approximately 260 °C (Figure 1a). Once again, it is evident that different alkaline earth metals play a critical role in the crystallization behavior of glass compositions. Nb*y*-Mg samples exhibited a monotonic decrease in Δ*T* as the Nb_2_O_5_ content increased from 10 to 20 mol %, while Nb*y*-Sr samples showed a monotonic increase in Δ*T* from 5 to 15 mol % of Nb_2_O_5_, with values ranging from approximately 150 to 250 °C.

Regarding the crystallization behavior, as shown in Figure 1b, it is evident that both the onset crystallization temperature (*T*_x_) and the maximum crystallization temperature (*T*_p_) increase with increasing Nb_2_O_5_ content for all samples, except for the *T*_x_ value for the Nb20–Ca sample, which will be discussed below. The different slopes observed for a set of samples containing different alkaline earth metals highlight the critical role of these cations in the glass crystallization trends.^44^ The exception observed for the Nb20–Ca sample is noticeable: *T*_p_ continues to increase relative to the Nb15–Ca sample, while *T*_x_ decreases from 767 to 718 °C. This can be interpreted as the formation of new crystalline phases at higher niobium contents. A crystallization study conducted by Stunda-Zujeva et al.^43^ demonstrated that lower concentrations of Nb_2_O_5_ favored the formation of calcium phosphate-based phase formation, while higher Nb_2_O_5_ contents promoted the crystallization of needle-like niobate or phosphoniobate phase crystallization. Therefore, it is plausible that in the Nb*y*-Ca glasses, the increased Nb_2_O_5_ content, combined with the significantly higher Δ*T*, led to the precipitation of new niobium-containing phases. This would explain the observed decrease in *T*_x_ without a corresponding decrease in *T*_p_ for the Nb20–Ca sample as well as why similar behavior was not observed for the Nb20–Mg and Nb20–Sr samples. These samples exhibited lower thermal stability and were thus less prone to forming niobate-based crystal phases, which require higher temperatures to precipitate.^43^ The presence of fluorides and the influence of alkaline earth cations (Mg^2+^, Ca^2+^, Sr^2+^, and Ba^2+^) significantly affect the network connectivity and the crystallization tendency. Cations with smaller ionic radii, such as Mg^2+^, promote greater structural connectivity and, consequently, higher thermal stability. In contrast, cations with larger radii, such as Ba^2+^, tend to inhibit crystallization. This behavior demonstrates the system’s remarkable ability to adjust its structure, resulting in thermal stability superior to that reported in the cited literature.^43^

UV–vis–NIR absorption spectroscopy was performed to investigate the absorption behavior of the glasses between 200 and 1000 nm. As Figure S5 shows, all samples exhibited similar absorption profiles with high transparency from approximately 350 to 1000 nm. The electronic absorption edge shifts monotonically toward higher wavelengths as the Nb_2_O_5_ content increases (corresponding to a decrease in the P/Nb ratio from 7.5 to 1.5).^13,36^ This shift suggests modifications in the energy levels of the valence and conduction bands, i.e., a reduction in the optical bandgap energy (*E*_g_), indicating that higher Nb_2_O_5_ concentrations enhance the covalency of the phosphate chains. This occurs through the incorporation of NbO_6_ octahedra, which replace P–O–P bonds with P–O–Nb and Nb–O–Nb bonds. The increase in covalency also correlates with an increase in the optical basicity of the glass network because the higher Nb_2_O_5_ content increases the number of nonbridging oxygens, primarily by altering the phosphate backbone chains.^45^ Across the different compositions, the same absorption behavior was observed regardless of the alkaline earth metal, except for the Nb*y*-Ba samples. Figure 2 illustrates the shift in the absorption edge for the Nb20-X samples. This shift is associated with a decrease in the ionic character of the glass network as the ionic radius of the alkaline earth metal decreases, accompanied by a tendency to lose valence electrons. This observation is consistent with the previously discussed trend in glass transition temperatures, where *T*_g_ decreases as the ionic radius of the alkaline earth cation increases.

**Figure 2:**
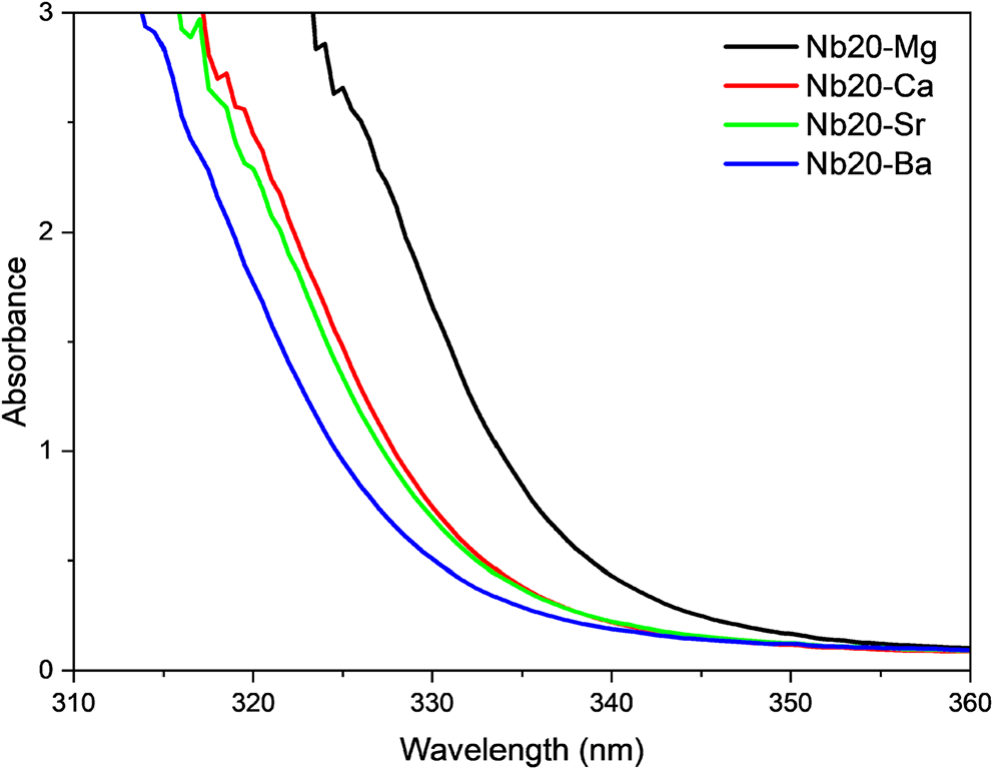
Absorption edge in the UV range for all Nb20-X samples, showing the shift based on different alkaline earth metals.

The optical bandgap value was calculated from the UV–vis–NIR absorption data. Optical transitions occur directly or indirectly between the valence and conduction bands within the optical bandgap. The optical bandgap relates to the molar absorptivity coefficient through the following equation:^46,47^

1 $$\alpha h\nu =B{\left(h\nu -E_{g}\right)}^{s}$$

in which *s* alternates between 1/2 for direct optical transitions and 2, for indirect transitions, *h* represents Planck’s constant, ν is the frequency, *B* is an energy-independent constant, *E*_g_ is the energy of the optical bandgap, and α is the linear absorption coefficient, calculated from the relation $\alpha =2.303\times \frac{A}{d}$ , where *A* is the absorbance and *d* is the thickness of the glass.^47,48^ The Urbach energy, related to the width of the band tail within conduction and valence bands, can be calculated by the equation:^46^

2 $$\alpha \left(\nu \right)=\alpha_{0}\exp\left(\frac{h\nu }{\Delta E}\right)$$

or by plotting ln­(α) against *h*ν, and its values and trends are frequently associated with the degree of defects within a disordered material.^49^ Figure S6 shows the graphic method used to obtain Urbach energy (*E*_U_) and the direct and indirect optical bandgap (*E*_dir._ and *E*_indir._) values, by plotting ln­(α), (α*h*ν)^2^, and (α*h*ν)^1/2^, respectively, as a function of the photon energy, *h*ν (eV). Extrapolating the linear portion of these curves (the region correlated with the absorption edge of the samples) provides the approximate values for the optical bandgap, shown in Figure 3. With the increase in Nb_2_O_5_ content (decrease in the P/Nb ratio), a higher covalency of the glass network is expected due to the NbO_6_ octahedra insertion, which strongly interacts within the phosphate units, increasing the number of NBO.^13,47^ This change is responsible for the observed red shift of the absorption edge in the samples, leading to a reduction in both the direct and indirect optical bandgap values, as shown in Figure 3a. Along with the decrease in *E*_dir._ and *E*_indir.,_ with increasing Nb_2_O_5_ molar concentration, the Δ*E* value also decreases from an average of 0.22 eV in Nb5-X samples to ∼0.17 eV in Nb20-X samples, as shown by the bars in Figure 3a. Figure 3b shows that increasing Nb_2_O_5_ concentration results in a decrease in the *E*_U_, suggesting that lower P/Nb ratios lead to a less disordered structure due to the formation of NbO_6_ ordered clusters.^49^ These trends were consistently observed in all synthesized samples, regardless of the alkaline earth metal. The Nb5–Ba sample, however, is not shown due to its anomalous behavior in the absorption spectra.

**Figure 3:**
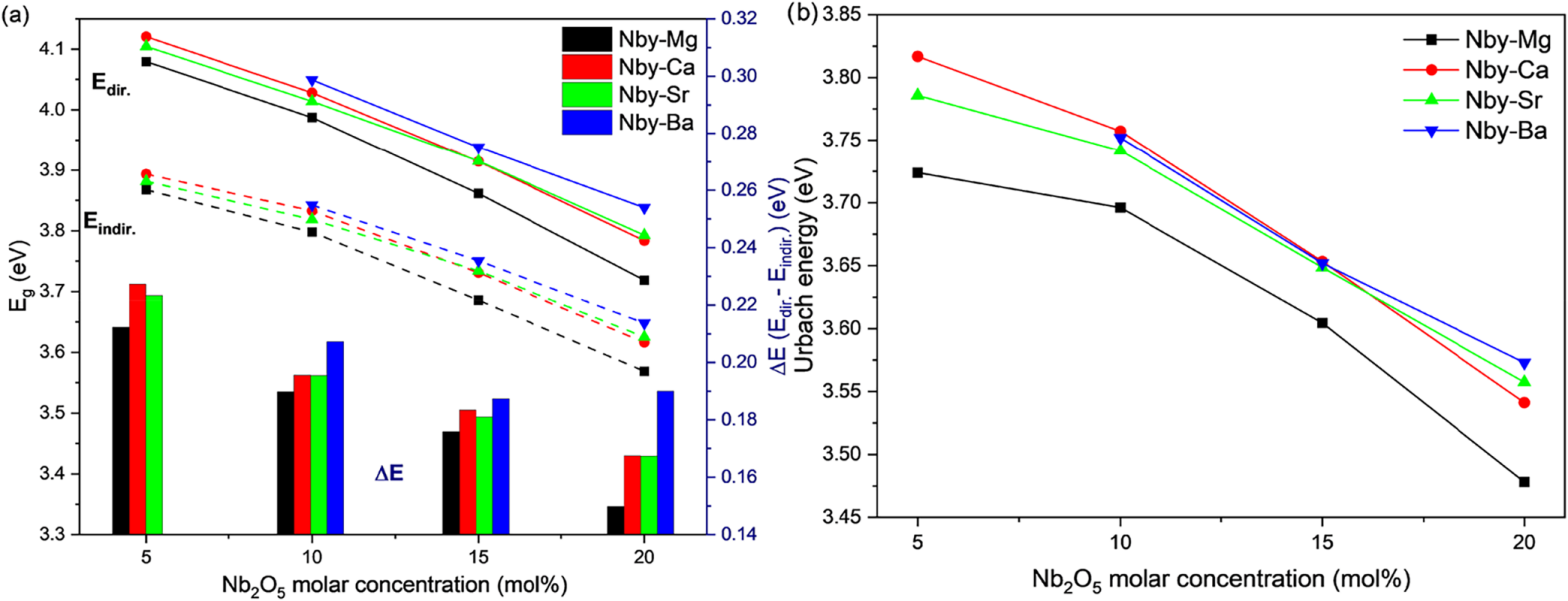
Direct and indirect optical bandgap energy values (*E*_dir._ and *E*_indir._), represented as solid and dashed lines (respectively) and symbols, and Δ*E* (*E*_dir._ – *E*_indir._), represented as bars (a), and Urbach energy calculated for all Nb*y*-X glass samples (b).

Unlike the clear monotonic shift in *E*_g_ values observed with varying Nb_2_O_5_ content, changes in the alkaline earth metal led to more complex behavior, as shown in Figure 3. Alkaline earth metals are known to be glass modifiers, acting as stabilizing nonbridging oxygens (NBOs) and linking phosphate–niobate chains through electrostatic interactions.^16^ Unlike Nb_2_O_5_, which can act as a network modifier or former depending on its concentration, variations in the alkaline earth metals provoke less pronounced shifts in the absorption edge, as well as in the *E*_g_ and *E*_U_ values, compared to the changes induced by variations in the P/Nb ratio. Several studies have correlated changes in various glass properties with their cationic potential (*Z*/*r* ratio).^19,50^ In this context, the variations observed in Figure 3, in which the Mg-containing samples exhibit significantly lower *E*_indir._, *E*_dir._, and *E*_U_ values, can be attributed to Mg^2+^ higher cationic potential compared with the other alkaline earth metals. Differences in optical bandgap values among the various alkaline earth metals become more pronounced with increasing Nb_2_O_5_ content. This suggests an enhancement of the covalent character of the mean glass linkages resulting from a decrease in alkaline earth metal ionic radius (or an increase in electronegativity/cationic potential), which increases in the presence of higher Nb_2_O_5_ concentrations. This behavior implies that the substitution of different alkaline earth metals primarily affects the niobate [NbO_6_] units incorporated into the phosphate backbone chains while exerting a comparatively smaller influence on the phosphate network itself. Supporting this conclusion, ^25^Mg NMR results indicate that the local coordination environment of the alkaline earth metal is strongly affected by the presence of [NbO_6_] units, as will be discussed later. Furthermore, for the Nb20-X samples, the optical bandgap values show a clear correlation with the electronegativity of the alkaline earth metals, exhibiting a decrease in *E*_g_ with increasing electronegativity, which varies as 0.89, 0.95, 1.00, and 1.31 for Ba^2+^, Sr^2+^, Ca^2+^, and Mg^2+^, respectively, according to Pauling’s electronegativity scale.^51^

Regarding the *E*_U_ values (Figure 3b), while the samples containing Ca^2+^, Sr^2+^, and Ba^2+^ display similar values with only minor variations, the Mg^2+^-containing glasses exhibit different behavior. The consistently lower *E*_U_ values observed for the Nb*y*-Mg glasses, regardless of Nb_2_O_5_ content, suggest a significantly lower degree of structural disorder. This behavior is attributed to the smaller ionic radius and higher cationic potential of Mg^2+^ compared to the other alkaline earth metals, which contribute to a decrease in their thermal stability against devitrification.

Raman spectroscopy was used to investigate the effect of varying Nb_2_O_5_ content and different alkaline earth metals on the structural role of the niobium-fluorophosphate glass network. As shown in Figure S7, spectra are grouped according to the alkaline earth metal used, and the individual curves within each group correspond to different Nb_2_O_5_ concentrations. As expected, variation in the P/Nb ratio significantly influenced the Raman spectra, primarily due to the intermediate role of Nb_2_O_5_ acting as both a network modifier and a former. This dual behavior enables Nb_2_O_5_ to directly modify the phosphate network by inserting NbO_6_ octahedra and promoting the formation of P–O–Nb and Nb–O–Nb linkages. Progressive increases in the Nb_2_O_5_ content result in noticeable changes in the spectrum, reflecting these structural modifications. While the most significant spectral changes are related to the Nb_2_O_5_ concentration, variations in the alkaline earth metal also led to noticeable structural modifications, as discussed further.

Figure 4 illustrates the changes in the Raman spectral features as a function of increasing the Nb_2_O_5_ concentration, using the Mg^2+^-containing sample set as a representative example. All characteristic bands observed in this spectrum are present in the other compositions, with differences primarily in the intensity and slight shifts in the position of the bands, addressed in the forward discussions. As previously discussed, the incorporation of NbO_6_ octahedra at higher Nb_2_O_5_ contents significantly alters the phosphate glass network. The vibrational mode assignments corresponding to each spectral feature are summarized in Table 2, using the notation P*^n^* to represent phosphate units with *n* bridging oxygens.^13,36,52^ The principal Raman bands, apart from the one at 910 cm^–1^, are located at 1024, 1047, 1150, and 1216 cm^–1^, and are attributed to the symmetric stretching of P^0^ units (PO_4_^3–^), symmetric stretching of P^1^ units (OPO_3_^2–^), symmetric stretching of P–O–P, and the stretching vibration of P–O–P in P^2^ units, respectively. A consistent decrease in the intensity of these bands with increasing Nb_2_O_5_ content indicates a progressive disruption of the phosphate network, particularly in involving the P–O–P bridges. This structural alteration is attributed to the formation of P–O–Nb bonds as NbO_6_ units are integrated into the glass network, a phenomenon further corroborated by the ^31^P nuclear magnetic resonance results.

**Figure 4:**
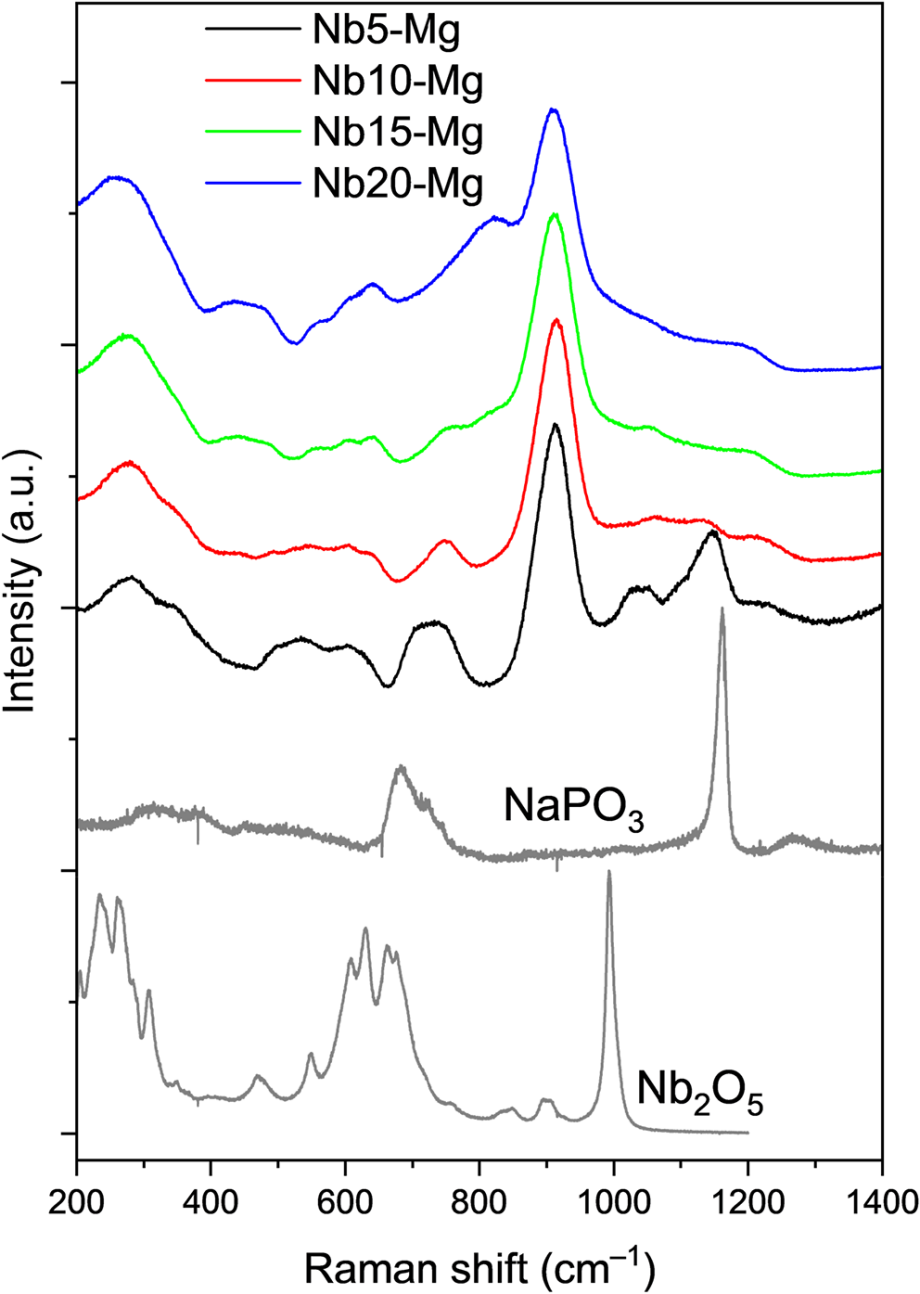
Raman spectra of the samples Nb*y*-Mg (*y* = 5, 10, 15, and 20 mol % of Nb_2_O_5_) and the precursors Nb_2_O_5_ and NaPO_3_.

**Table 2:** Main bands seen on the Raman spectra of Nb*y*-X samples (approximate values in cm^–1^ as same bands show different positions as a function of X and *y*) and attribution to molecular modes

Wavenumber (cm^–1^)	Assigned mode	References
276	O–P–O + O–Nb–O coupled deformation mode	^13,52,53^
430	O–P–O + O–Nb–O coupled mode	^13,52,53^
641	Nb–O vibrations	^13,52–54^
754	P–O–P symmetric bonding	^13,52,53^
824	Nb–O–Nb deformation mode	^13,52–54^
912	Nb–O short bond in NbO_6_	^13,52,53^
1024	P^0^ (PO_4_^3–^) symmetric stretching vibration	^13,52,53^
1047	P^1^ (OPO_3_^2–^) symmetric stretching vibration	^13,52,53^
1150	P–O–P symmetric vibration	^13,52,54^
1216	P^2^ P–O–P stretching vibration	^47,48^

Although P–F bonds can be weakly detected in fluorophosphate glass around 700 and 860 cm^–1^, as previously reported by Möncke and Eckert, these possible bands are overlapped by the broad and highly intense niobate bands, particularly the one centered at 824 cm^–1^.^4^ A similar effect was previously reported by da Silva et al. in lead pyrophosphate glasses with compositions Pb_2_P_2_O_7_–Nb_2_O_5_–XF_2_ (where X = Mg, Ca, Sr, and Ba),^55^ in which P–F bonds were also undetectable by Raman spectroscopy. To reliably assign these bonds, ^19^F NMR measurements were performed, and the results are presented on the following pages.

As the concentration of Nb_2_O_5_ increases, the intensity of the bands located at 276, 430, 641, and 824 cm^–1^—which are attributed to Nb–O bonds in different distorted NbO_6_ octahedra—increases as well.^13,48^ As previously reported, the H–Nb_2_O_5_ polymorph is the most stable form under the high-temperature conditions employed during glass synthesis (above 1000 °C).^13^ The Nb–O–Nb bending mode at ∼250 cm^–1^ is indicative of the clustering of NbO*_n_* units within the phosphate network.^13^ The increasing intensity of these low-frequency bands with a higher Nb_2_O_5_ content suggests the formation of niobate-rich domains, which is relevant to understanding the crystallization behavior. These results confirm that higher Nb_2_O_5_ concentrations promote substantial structural reorganization of the glass network’s structure, primarily by disrupting the phosphate backbone and forming new Nb–O linkages. Furthermore, the substitution of different alkaline earth metals also induces significant structural variations, as evidenced by changes in the Raman spectra.

Figure 5 presents the differences in the Raman spectral band profiles for glasses containing 5 and 20 mol % of Nb_2_O_5_ with different alkaline earth modifiers. The most prominent change observed in the Raman spectra bands is the systematic shift of vibrational bands to lower wavenumber values with an increasing ionic radius of the alkaline earth metal. In Figure 5a, the main band at approximately 900 cm^–1^ progressively shifts from 912 to 900 cm^–1^ when the alkaline earth metal periodically changes from Mg^2+^ to Ba^2+^. A similar trend is evident in Figure 5b, where the band shifts from 907 to 894 cm^–1^ across the same series, averaging a downshift of approximately 4 cm^–1^ per metal. This red shift reflects a periodic structural response induced by the incorporation of increasingly larger cations into the glass matrix. As reported in the literature, alkaline earth metals occupy positions between the niobium-phosphate chains within the glass network and exert electrostatic interactions with nonbridging oxygens.^16^ The observed shift in the Raman bands is attributed to the elongation of P–O and Nb–O bonds, likely caused by the accommodation of larger cations, which perturb the local bonding polarizability environment.^52^ This effect also correlates with the decrease in *T*_g_ as the ionic radius of the alkaline earth metal increases.

**Figure 5:**
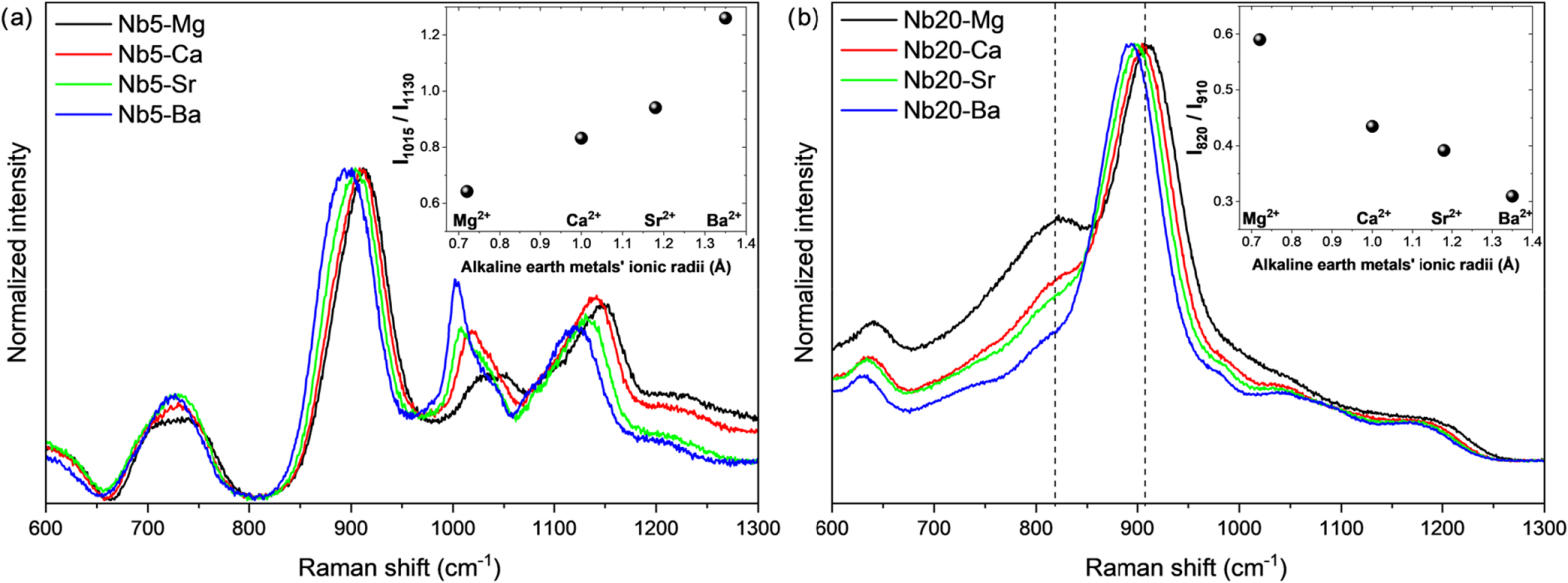
Raman spectrum of glass samples (a) Nb5-X and (b) Nb20-X in a shorter Raman shift range (cm^–1^), demonstrating the most noticeable differences caused in the spectra due to the different alkaline earth metals contained in the samples. Inset figures exhibit (a) the intensity ratios of the bands at ∼1015 cm^–1^ by the bands at ∼1130 cm^–1^ (*I*_1015_/*I*_1130_) and (b) the intensity ratios of the bands at ∼820 cm^–1^ by the bands at ∼910 cm^–1^.

In addition to the wavenumber shifts, variations in the relative intensities of specific Raman bands are also evident. As shown in Figure 5a, notable changes are observed in the bands centered around 1015 and 1130 cm^–1^, assigned to symmetric stretching vibrations of P^0^ and P^1^ phosphate units, respectively. The increase in the relative intensity of the P^0^ compared to the P^1^ band, as shown in the inset ratio *I*_1015_/*I*_1130_, suggests that larger alkaline earth cations promote the formation of more isolated phosphate tetrahedra. This indicates a higher degree of depolymerization within the phosphate network. These findings are consistent with observations from DSC and ^31^P MAS NMR measurements and suggest that the incorporation of larger alkaline earth metals results in a less connected glass structure.

Figure 5b shows that the higher niobium content in the Nb20-X glass samples reduces the prominence of phosphate-related Raman bands, particularly those above 1000 cm^–1^. This is primarily due to the strong polarizability of niobium-containing structural units. Nevertheless, a clear trend is evident in the relative intensities of bands at ∼820 and ∼910 cm^–1^. The band at ∼820 cm^–1^, attributed to the asymmetric deformation of Nb–O–Nb linkages in edge-sharing NbO_6_ octahedra forming chain-like structures, decreases in intensity relative to the band at ∼910 cm^–1^, which corresponds to the symmetric stretching of Nb–O bonds in isolated NbO_6_ units.^52,54^ The *I*_820_/*I*_910_ ratio is depicted in the inset of Figure 5b. This behavior suggests that alkaline earth metals with higher cationic potential and smaller ionic radii (e.g., Mg^2+^) favor the clustering of NbO_6_ units and promote the formation of niobate chains. In contrast, cations with a lower cationic potential (e.g., Ba^2+^) tend to stabilize more isolated NbO_6_ units. The observed variations in the 820/910 cm^–1^ intensity ratio reflect the modifying effect of the alkaline earth metal on the glass structure by modulating the organization of both phosphate and niobate units according to periodic trends in charge/radius ratio and electronegativity.

For the sake of clarity, the notation P_*m*_^*n*^ will be adopted in this section to describe phosphate structural units, where *n* denotes the total number of bridging oxygens (BOs) and *m* represents the number of P–O–Nb linkages. This classification provides a more precise description than the conventional Q*^n^* notation when discussing heteroatomic environments involving Nb–O–P bonds. Figure S8 shows the ^31^P MAS NMR spectra for all sample sets. Resonances for the Nb*y*–X glasses appear within the range of approximately 0 to −30 ppm. While the spectra are remarkably similar across different alkaline earth metals, more pronounced changes are observed with an increasing Nb_2_O_5_ content. For glasses containing 5 mol % Nb_2_O_5_, the spectra exhibit a dominant resonance at −8 ppm, accompanied by a less intense signal near −20 ppm. As the Nb_2_O_5_ concentration increases to 10, 15, and 20 mol %, the −20 ppm peak progressively decreases and eventually vanishes. Meanwhile, the broader signal around −8 ppm shifts toward higher chemical shift values.

To enhance the spectral resolution and better elucidate the magnetic environments of phosphorus nuclei, additional ^31^P NMR measurements were performed using different pulse sequences. While Figure S8 illustrates the patterns obtained using the single-pulse sequence, Figure 6 compares spectra from the Nb*y*-Ca sample set obtained using both single-pulse and refocused INADEQUATE (INAD) techniques.^23^ The INAD sequence, which relies on the excitation and detection of double-quantum coherence, enables the selective observation of ^31^P species engaged in homonuclear dipolar couplings, particularly those involved in P–O–P linkages. The whole Nb*y*-Ca sample set is shown to demonstrate how different Nb_2_O_5_ concentrations influence the ^31^P NMR spectra. Also, samples Nb5–Mg and Nb20–Mg were measured (as shown in Figure S9a comparing the single-pulse and INAD experiments) to demonstrate the high similarity between samples containing different alkaline earth metals, showing that the prominent effect is caused by the Nb_2_O_5_ in this case and not the different modifier metal contained in the glass.

**Figure 6:**
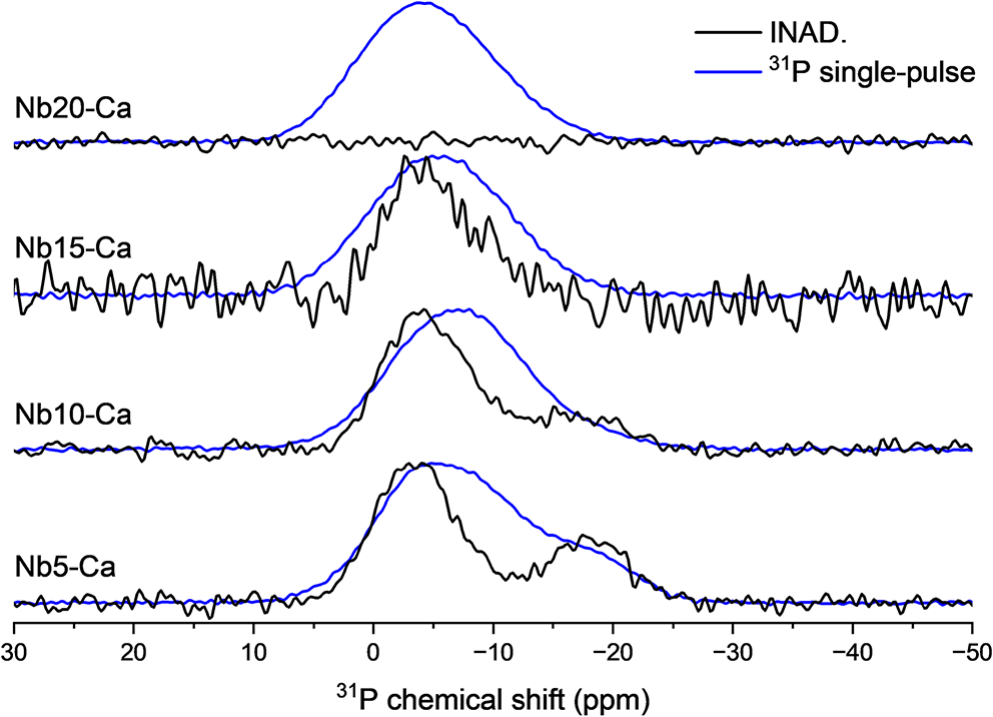
Nuclear magnetic resonance spectrum of the Nb*y*-Ca sample set (*y* = 5, 10, 15, and 20 mol % of Nb_2_O_5_), monitoring the ^31^P, with experimental data from the single-pulse and refocused INADEQUATE spin–echo (INAD) experiments.

After acquiring the single-pulse and INAD ^31^P NMR spectra, deconvolutions of the single-pulse spectra were carried out using the chemical shift positions and line widths obtained from the INAD spectra as fixed parameters. This approach enables the deconvolution of the missing bands by comparing the INAD peaks to the total spectrum obtained by one pulse, facilitating the identification of each distinct resonance peak. The deconvolution of the spectrum is shown in Figure 7 for the Nb*y*-Ca samples containing 5, 10, 15, and 20 mol % of Nb_2_O_5_, while the deconvolution of Nb5–Mg is shown in Figure S9b. The spectra related to the sample Nb20–Ca were not deconvoluted in the same way due to the nondetectable signal with the INAD experiment. Raman spectroscopy reveals that P–O–P linkages are present for this sample (between 1500 and 1220 cm^–1^), and Figure S10 depicts the high similarity between samples Nb15–Ca and Nb20–Ca, mainly above 950 cm^–1^ (phosphate groups, as assigned in Table 2). Hence, the lack of signal in the INAD experiment is probably caused by very short spin–spin transversal relaxation times due to strong dipolar interactions with ^93^Nb. Therefore, information about P–O–P connectivity is not definitive from solid-state NMR alone. Hence, the deconvolution for Nb20–Ca was performed by using the same parameters (peak location and width, in parts per million) as used for Nb15–Ca, and the relative areas were tentatively calculated (marked with an asterisk in Table 3).

**Figure 7:**
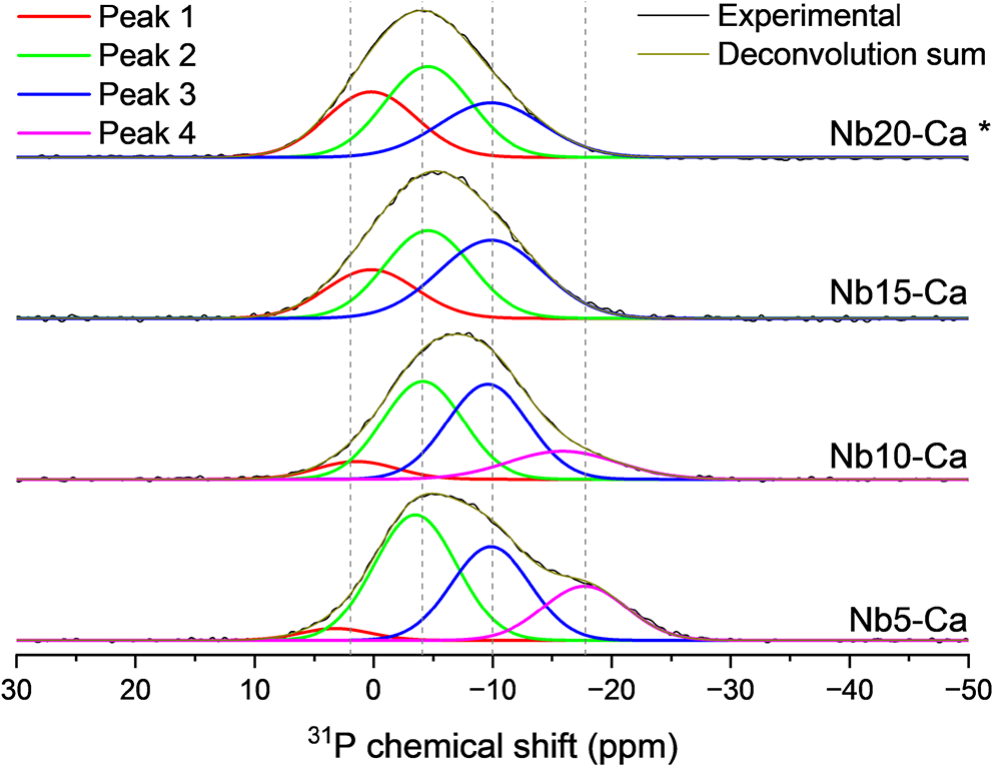
Nuclear magnetic resonance spectrum of the Nb*y*-Ca series of samples (*y* = 5, 10, 15, and 20 mol % of Nb_2_O_5_), monitoring the ^31^P nucleus, with deconvolution of the bands from the refocused-INADEQUATE spin–echo (INAD) experiment, with rotation at 15 kHz. Nb20–Ca is marked with an asterisk (*) due to the nonappearance of the INAD signal. Deconvolution was based on Nb15–Ca’s peak position and width.

**Table 3:** Assigned phosphate groups and relative areas related to each peak (in %) from the deconvolutions of Nb*y*-Ca samples (*y* = 5, 10, 15, and 20 mol % of Nb_2_O_5_), in Figure 7

		Relative area (%)			
Assigned phosphate groups	Chemical shift (ppm)	Nb5–Ca	Nb10–Ca	Nb15–Ca	Nb20–Ca*^a^
P^0^ + P_1Nb_^1^	0.2 to 3.2	3	7	22	30
P^1^	–3.5 to −4.5	45	39	38	40
P_2Nb_^2^	–9.7 to −9.8	32	39	40	30
P_1Nb_^2^ + P_0Nb_^2^	–15.9 to −17.7	20	15	0	0

a Nb20–Ca relative areas were tentatively calculated based on the deconvolution with the same peak parameters as Nb15–Ca.

As indicated by the dashed lines over the spectra in Figure 7, by using this double-quantum filtering technique approach, we could identify four spectral components at approximately −17, −10, −4, and 2 ppm. Table 3 shows the areas corresponding to each peak observed from the deconvolution of the ^31^P NMR spectra, the variations in the peak center, and the assigned phosphate groups (Figure 7), while Table S2 shows these variables related to the deconvolution of Nb5–Mg glass (Figure S9b). The components at −17 and −4 ppm are present in the INAD spectra, indicating that they are assigned to phosphate groups containing P–O–P bonds. In contrast, the components located at −10 and 2 ppm are absent in the INAD spectra, indicating that they do not participate in P–O–P linkages. Therefore, these components can be assigned to only one of the following species: P^0^, P_1Nb_^1^, or P_2Nb_^2^ (P_3Nb_^3^ is ruled out by Raman data). Considering the chemical shift information,^56^ compositional variations, and ^31^P­{^93^Nb} RESPDOR data described below, we attribute the resonance at 2 ppm to mixed contributions from P^0^ and P_1Nb_^1^ units and the component in −10 ppm to P_0Nb_^1^ units. On the other hand, also based on the literature and on RESPDOR data, assignments can be found for the resonances at −17 and −4 ppm.^56^ The line at −17 ppm can be attributed to a mixture of P_0Nb_^2^ and P_1Nb_^2^ (P_0/1Nb_^2^), with an increasing contribution of P_1Nb_^2^, as indicated by the broadening and low-field shift of this line (RESPDOR data show that this low-field shift is expected for higher *m* values in P_*m*Nb_^n^ units). Finally, the component at −4 ppm is attributed to the P_0Nb_^1^ species.

With increasing Nb_2_O_5_ concentration, there is a clear decrease of the ^31^P resonance corresponding to P_0/1Nb_^2^, with the complete disappearance of this peak in 15% Nb_2_O_5_. The decrease in this component is followed by a decrease in P_0Nb_^1^ (−4 ppm) and an increase in the concentration of P_2Nb_^2^ (−10 ppm) and P_1Nb_^1^ and P^0^ species (2 ppm). This behavior underscores the formation of P–O–Nb and Nb–O–Nb bonds to the detriment of the P–O–P bond.^57,58^ These alterations, also discussed using DSC and Raman spectroscopy techniques, are reiterated by the NMR results.^52,54,59^ Also, similar behavior regarding the progressive alteration of the phosphate groups by decreasing the P/Nb molar ratio was previously reported for glasses based only on Na_2_O, P_2_O_5_, and Nb_2_O_5_.^36,54^

Figure 8 presents the overlaid ^31^P NMR spectra to illustrate how the resonance peaks shift with the incorporation of different alkaline earth metals into the glass matrix. While the overall spectral pattern remains consistent across samples containing different alkaline earth metals, it undergoes significant changes with varying Nb_2_O_5_ concentrations. A closer comparison of spectra for samples with the same Nb_2_O_5_ content but different alkaline earth metals (Figure 8a) reveals a progressive high-field shift of the main resonance peak, which correlates with the cationic potential (*Z*/*r*) of the modifying cation (2.78, 2.00, 1.72, and 1.36 Å^–1^ for Mg^2+^, Ca^2+^, Sr^2+^, and Ba^2+^, respectively). Alkaline earth metals with smaller ionic radii, such as Mg^2+^ and Ca^2+^, exhibit higher cationic potentials, resulting in a more pronounced shift of the ^31^P NMR resonance peaks toward lower chemical shift values (higher shielding). This effect is attributed to the periodical increase in the covalent character of the interaction between the alkaline earth cations and the NBOs from Ba^2+^ to Mg^2+^, which enhances the electronic shielding of the phosphorus nuclei bonded to these NBOs.^56,57,60^

**Figure 8:**
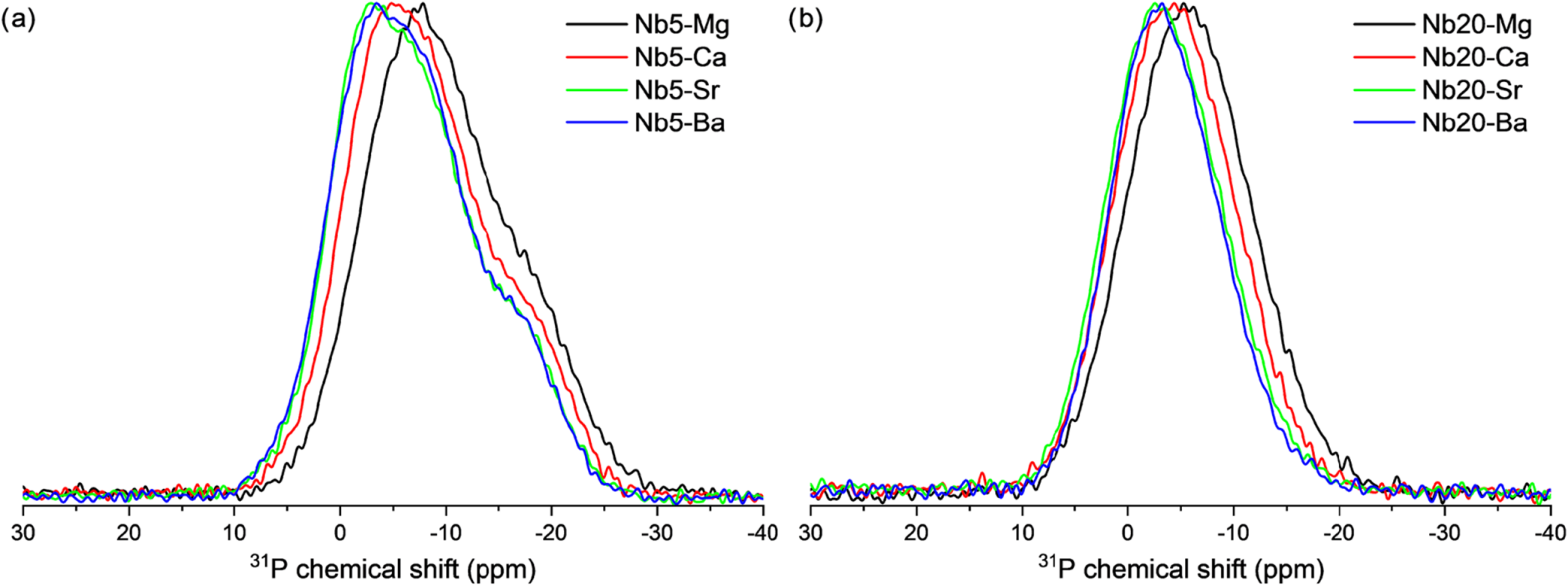
Spectra of nuclear magnetic resonance curves, monitoring the ^31^P of samples (a) Nb5-X and (b) Nb20-X, rotated at 15 kHz.

The ^23^Na NMR spectra for all 16 Nb*y*-X glass samples are shown in Figure S11. All spectra display a single broad resonance centered at around −14 ppm, corresponding to sodium ions in a disordered glassy environment. Notably, a sharp signal at −5.4 ppm is observed in the spectra of the Ca^2+^, Sr^2+^, and Ba^2+^ samples with 5 mol % of Nb_2_O_5_, indicating the presence of a partially crystallized phase. This effect is not observed in the Mg-containing glass, suggesting that partial crystallization is dependent on the nature of the alkaline earth metal. The low Nb_2_O_5_ content increases the hygroscopicity of the glasses, making them more prone to absorbing atmospheric moisture.^61^ This behavior is consistent with the loss of transparency observed in bulk samples containing 5 mol % of Nb_2_O_5_, except for the Nb5–Mg glass, which maintained its transparency since synthesis—corroborating the findings from the ^23^Na NMR results.

Direct comparisons of the ^23^Na spectra are shown in Figure S12, as a function of Nb_2_O_5_ concentration for the Nb*y*-Mg system (Figure S12a), and as a function of alkaline earth metal species samples containing 20 mol % of Nb_2_O_5_ (Figure S12b). Only minor, though systematic, chemical shift variations are observed, indicating that the alkaline earth cations and Nb-containing units primarily influence the second coordination sphere of the Na^+^ environment. This interpretation is supported by the ^23^Na­{^31^P} REDOR^62,63^ results, shown in Figure S13 of the Supporting Information, which demonstrate that the ^23^Na–^31^P coordination environment remains largely consistent across all samples.

^31^P­{^23^Na} REAPDOR experiments were also obtained only for the Nb*y*-Mg glass series, and the resulting dephasing curves are shown in Figure 9 in comparison to the reference curve for the Na_2_PO_3_F crystal. Similar to the ^23^Na­{^31^P} REDOR experiments, these results show that even when altering the Nb*y*-Mg glasses from 5 to 20 mol % of Nb_2_O_5_, the difference in the dephasing between phosphorus and sodium nuclei is not significant, showing a similar behavior of Δ*S*/*S*_0_ in function of the time. Also, Table S3 depicts the calculated values for experimental second moments (*M*_2(Na–P) exp._) and the calculated number of phosphorus (*N*_P_), demonstrating similar behavior for the whole Nb*y*-X glass sets. This confirms that the phosphorus environment is also not changing, in terms of the amount and proximity of sodium species, when altering the Nb_2_O_5_ molar concentration. To further analyze how the ^31^P and ^23^Na interaction induces changes onto the ^31^P NMR spectra, Figure 10 compares the *S*_0_, *S*, and Δ*S* for samples Nb5–Mg (Figure 10a,b) and Nb20–Mg (Figure 10c,d) at different times of evolution, being 0.5 ms (Figure 10a,c) and 0.8 ms (Figure 10b,d).

**Figure 9:**
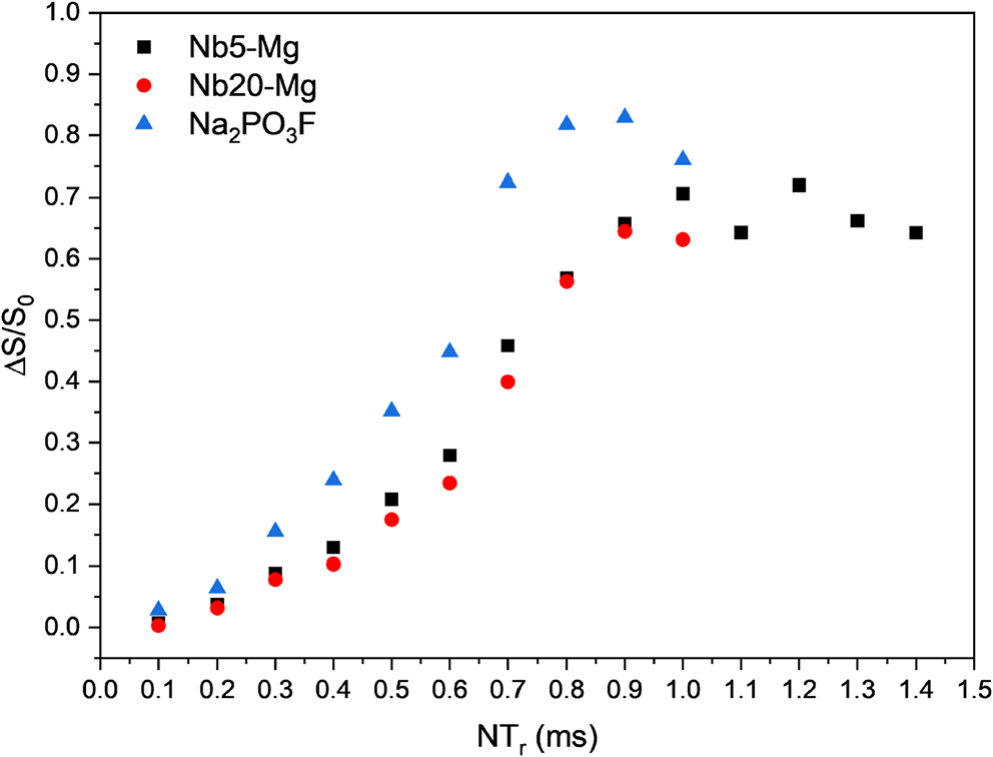
^31^P­{^23^Na} REAPDOR dephasing curves obtained for Nb5–Mg and Nb20–Mg glass samples, along with the reference measured for the Na_2_PO_3_F crystal.

**Figure 10:**
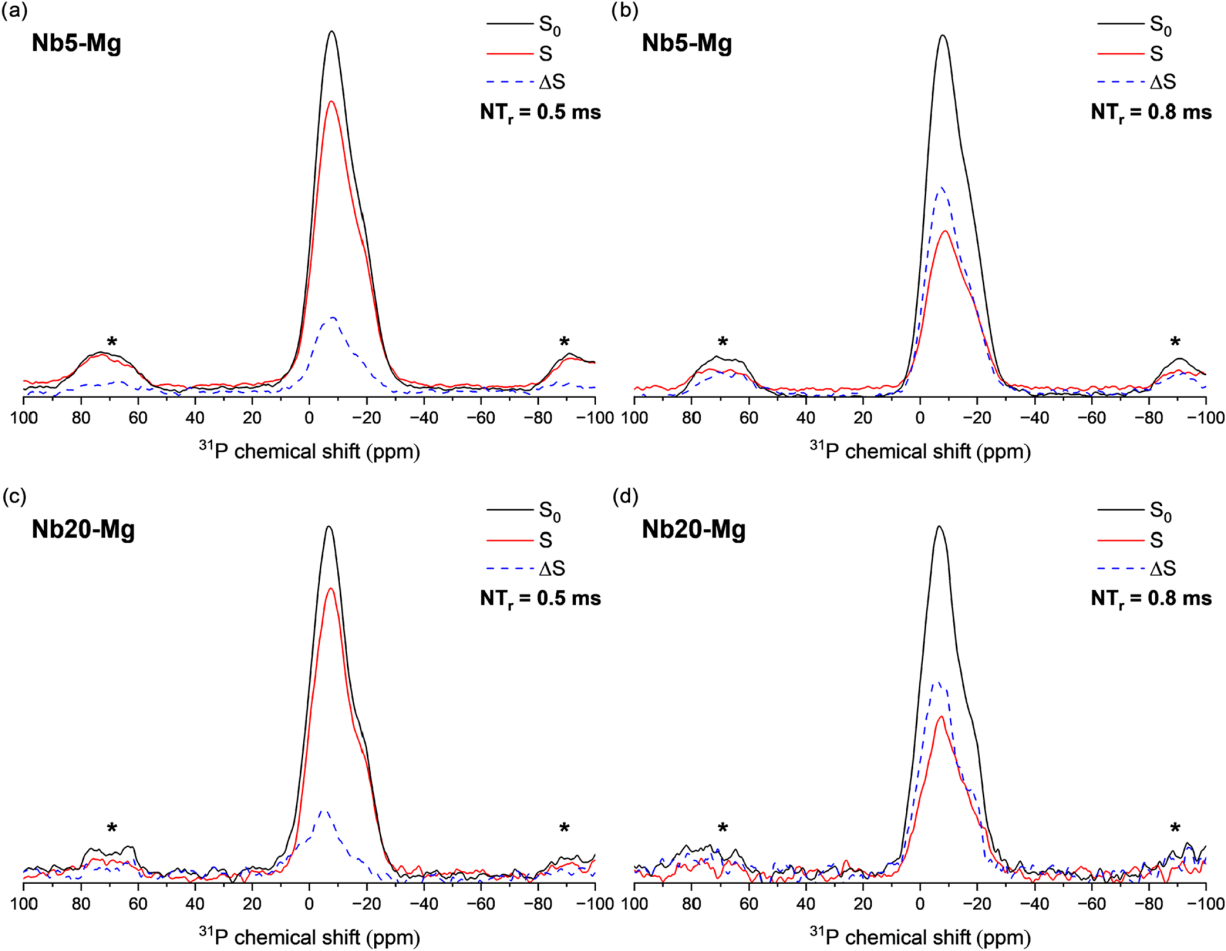
^31^P­{^23^Na} REAPDOR results obtained for Nb5–Mg (a, b) and Nb20–Mg (c, d) glass samples, depicting the *S*_0_ (black curve), *S* (red curve) and Δ*S* = *S*_0_ – *S* (dashed blue curve) for ten rotor cycles (500 μs) (a, c) and 16 rotor cycles (800 μs) (b, d).

The comparison presented in Figure 10 demonstrates that the ^31^P nuclei resonating near −4 ppm are interacting most strongly with the ^23^Na nuclei. The shape of the Δ*S* curve remains consistent across different samples for a given evolution time and also when comparing the same sample at different evolution times. The consistency with the REAPDOR effect suggests that no considerable differentiation in the Na–P interactions occurs with the explored variables in the studied glass compositions. As expected, increasing the evolution time from 0.5 to 0.8 ms leads to a higher Δ*S* intensity, reflecting an increased dipolar coupling between the ^31^P and ^23^Na nuclei. However, the similar intensity changes observed across samples further support the conclusion that variations in the Nb_2_O_5_ content do not substantially affect the coordination environment between sodium and phosphorus.

^31^P­{^93^Nb} RESPDOR experiments were conducted mainly to visualize how the recoupling of the ^93^Nb dipolar interaction would suppress the ^31^P magnetic resonance, depending on the time (amount of rotor cycles) and on the Nb_2_O_5_ concentration in the glass. Figure 11 depicts the Δ*S*/*S*_0_ dephasing as a function of time, for up to 16 rotor cycles (400 *μs*), for samples Nb10–Mg and Nb20–Mg. Qualitative information can be obtained from these curves. The RESPDOR dephasing is more pronounced for the sample containing a higher concentration of Nb, confirming our previous attributions regarding the formation of Nb–O–P heterolinkages.

**Figure 11:**
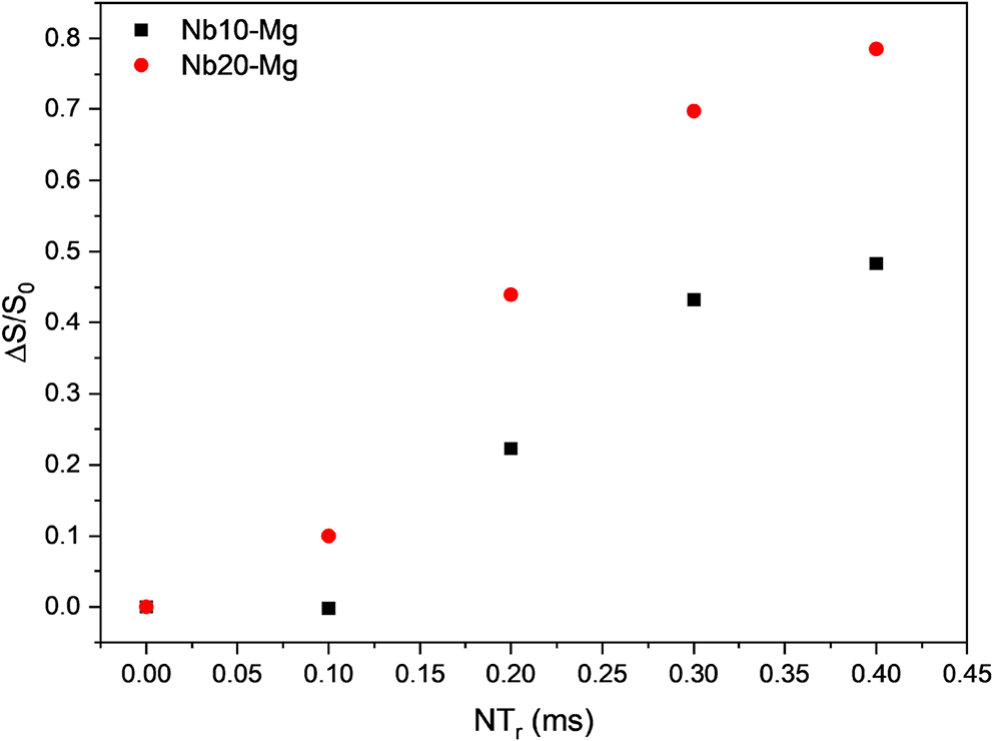
^31^P­{^93^Nb} RESPDOR dephasing curves obtained for the Nb10–Mg and Nb20–Mg glass samples.

Figure 12 shows the comparison between RESPDOR ^31^P spectra acquired with (*S*) and without (*S*_0_) the application of the dipolar recoupling scheme on the ^93^Nb channel. We have selected the spectra for two different evolution times: 4 rotor cycles (200 μs) (Figure 12a,c) and 8 rotor cycles (400 μs) (Figure 12b,d). The difference spectra (Δ*S* = *S*_0_ – *S*) are also displayed (dashed curves). The comparison between the RESPDOR *S* and *S*_0_ spectra reveals that there is an overall dephasing of the spectra, meaning that all ^31^P species are, to some extent, dipolarly coupled to ^93^Nb. This reveals the homogeneous NbO_6_ units’ distribution in the phosphate network. The Δ*S* curves are slightly shifted to higher chemical shift values, indicating that the replacement of a P–O–P for a P–O–Nb linkage shifts the ^31^P resonance to a lower field. This observation agrees with our above assignments.

**Figure 12:**
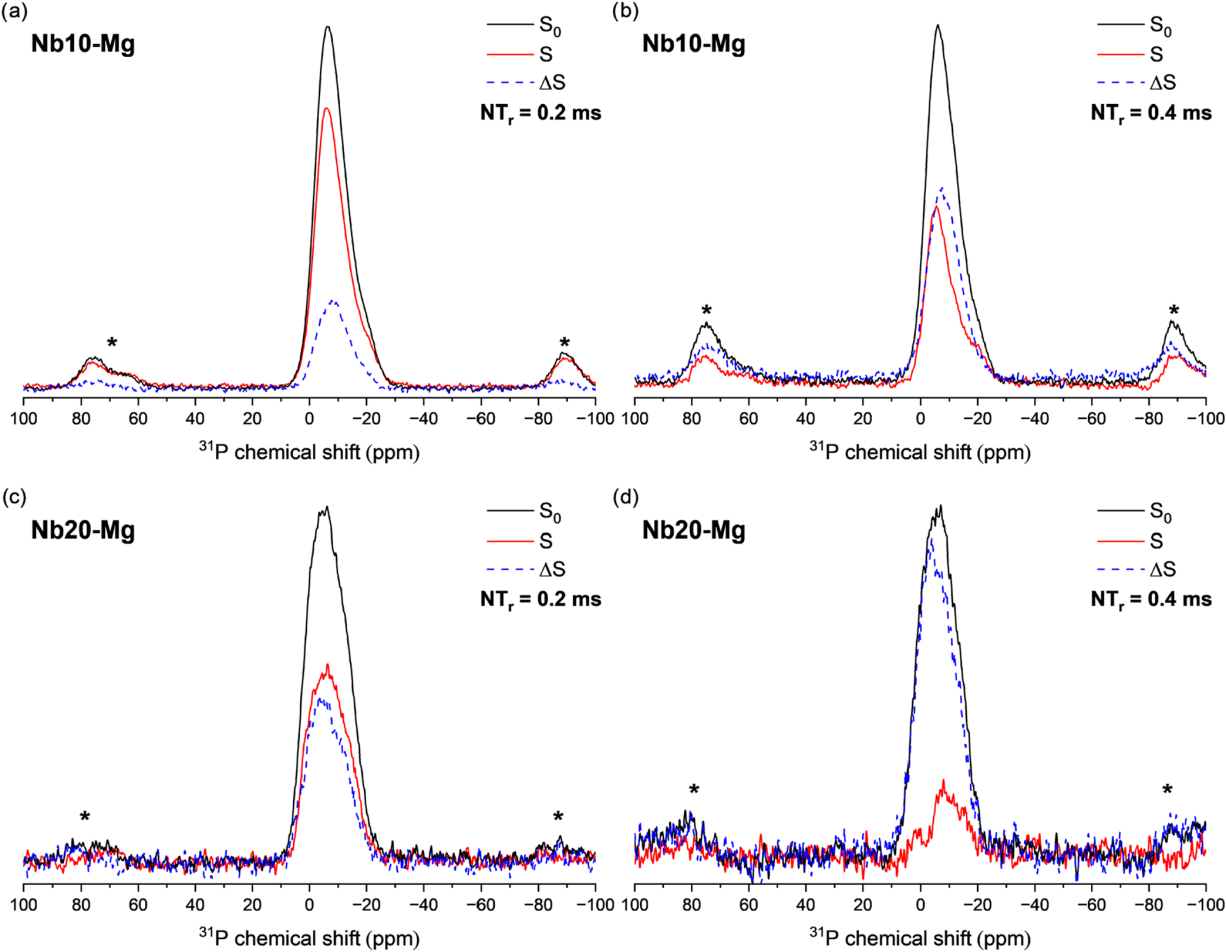
^31^P­{^93^Nb} RESPDOR results obtained for Nb10–Mg (a, b) and Nb20–Mg (c, d) glass samples, depicting the *S*_0_ (black curve), *S* (red curve) and Δ*S* = *S*_0_ – *S* (dashed blue curve) for four rotor cycles (200 μs) (a, c) and eight rotor cycles (400 μs) (b, d).

Figure 13 shows the ^19^F MAS NMR spectra for the investigated glasses. The spectral features are highly dependent on the alkaline earth metal and the concentration of Nb_2_O_5_. Specifically, the resonance of fluorine coordinated with phosphorus, P–F (located around −75 ppm),^64^ is quite pronounced in samples containing between 5 and 15 mol % of Nb_2_O_5_. The ^31^P­{^19^F}-REDOR technique could provide crucial insights for assigning the P–F bond to its specific tetrahedral [PO_*x*_F_4–*x*_] unit. However, the required probe for this experiment is not currently available in our facilities, and the experiment will be performed soon. For all systems, as the concentration of Nb_2_O_5_ increases, the relative intensity of these peaks decreases, indicating that the formation of P–F bonds becomes less probable when compared to the F coordination with Nb and alkaline earth species. In contrast, peaks located between −25 and −40 ppm, and between −120 and −135 ppm, become more prominent with higher concentrations of Nb_2_O_5_, particularly in samples with 20 mol % Nb_2_O_5_. These peaks are attributed to Nb–F bonds, indicating that the increase of Nb_2_O_5_ concentration noticeably increases the fluorine coordination with niobium.^55,65^ This trend highlights how the incorporation of Nb_2_O_5_ influences the bonding environment of fluorine within the glass matrix, altering both the structural and optical properties of the material.^65^

**Figure 13:**
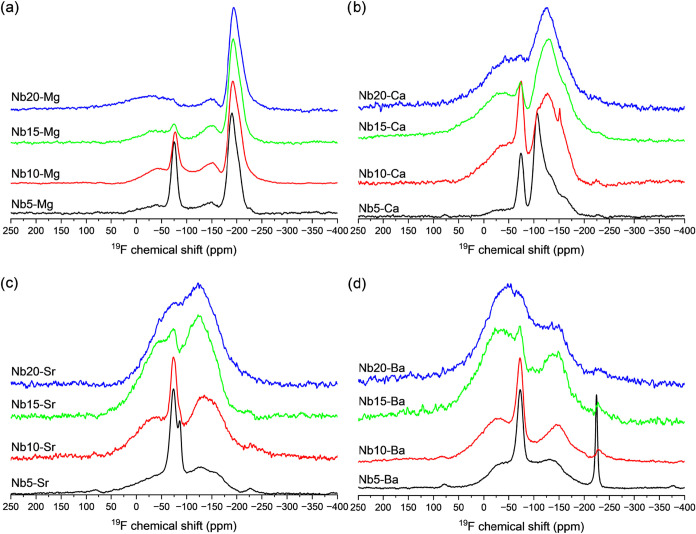
Set of nuclear magnetic resonance (NMR) spectra, monitoring the ^19^F of Nb*y*-X samples, rotated at 35 kHz, with X = (a) Mg^2+^, (b) Ca^2+^, (c) Sr^2+^, and (d) Ba^2+^.

In their study of ^19^F NMR in various oxyfluoride compounds, Du et al. observed that the displacement of resonances related to Nb–F bonds varies significantly. These variations are influenced not only by the central metal (niobium, in the case of this study) and the cations surrounding the Nb–O–Nb chains, and the presence of other fluorides coordinated to the same Nb atom.^65^ The broad width of the Nb–F bands can be attributed to the unresolved quadrupolar coupling of the neighboring ^93^Nb nuclei and the positioning of the fluorine nuclei either above or below the Nb–O–Nb chains.^65^ The Nb5–Ba sample was the only one to exhibit a very narrow and intense peak at approximately −223 ppm, likely indicating the formation of the Na–F bond.^66^ The appearance of this peak at approximately −223 ppm in the Nb5–Ba sample is associated with the high hygroscopic character of this sample, suggesting that crystallization induced by the absorption of atmospheric water led to the formation of Na–F bonds. Regarding the fluorine coordination with different alkaline earth metals, the periodic variation in the ionic radii of each metal and their cationic potential also determines how these bonds are formed within the glass. For instance, the Ba–F bond (expected at −14 ppm) was not detected for any of the Nb*y*-Ba samples. While the Sr–F bond (−85 ppm) was found only for the composition containing 5 mol % of Nb_2_O_5_, the Ca–F bond (−107 ppm) was found in the 5, 10, and 15 mol % Nb_2_O_5_ samples.^67^ Exceptionally, Nb*y*-Mg samples not only clearly showed a Mg–F bond (−192 ppm) for all of the Nb*y*-Mg glasses but also exhibited this peak as the strongest among all fluorine coordination. Therefore, the variable cationic potential between different alkaline earth metals also plays a key role in the coordination with fluorine within the glass and, consequently, rules how the fluorine will be able to coordinate with other species.^67^

The ^19^F NMR results indicate that fluorine coordination exhibits more pronounced changes compared to that of the ^31^P nucleus, which is influenced by both the niobium concentration and the type of alkaline earth metal. For instance, the high hygroscopicity of the Nb5–Ba sample resulted in crystallization and the formation of Na–F bonds. This was also detected by ^23^Na NMR and confirms the earlier assumption that interaction with atmospheric moisture leads to the development of more crystalline domains. Furthermore, the data support the hypothesis that fluorine preferentially coordinates with niobium at higher Nb_2_O_5_ concentrations, as evidenced by the increased intensity of Nb–F signals with rising niobium content. The results also reinforce the notion that fluorine exhibits a greater affinity for metals with smaller ionic radii. This is demonstrated by the absence of Ba–F bonds, the increasing presence of Sr–F and Ca–F bonds, and the particularly intense Mg–F signal, which remains strong regardless of the Nb_2_O_5_ concentration. This behavior indicates that fluorine preferentially coordinates with smaller cations, such as magnesium.

Finally, ^25^Mg MAS NMR spectra were acquired for samples Nb5–Mg and Nb20–Mg (Figure 14). The ^25^Mg spectral lineshapes are dominated by the quadrupolar coupling interaction and are characterized by a broad distribution of electric field gradients at Mg coordination environments, as typically found for ^25^Mg NMR in glasses.^68–75^ The lineshapes for both samples could be simulated by using a Czjzek model, which considers a multivariate normal distribution of quadrupolar coupling tensor parameters *C*_Q_ and η around an isotropic tensor.^76^ The Czjzek model is characterized by the parameter σ, which corresponds to the standard deviation of the EFG distribution.^76,77^ Both spectra were fitted with a single Czjzek component, yielding the following parameters: Nb5–Mg: δ_iso_ = 1.7 ppm, σ = 2.3 MHz; Nb20–Mg: δ_iso_ = −1.4 ppm, σ = 3.5 MHz. It is noteworthy that the isotropic chemical shifts and the Czjzek distribution widths (σ) reported here are significantly smaller than those commonly observed in oxide glasses,^78–80^ which may indeed be a consequence of the stronger and more ordered Mg–F interactions in this fluoride-rich glass matrix. The larger σ value for Nb20–Mg suggests that Nb incorporation leads to a broader EFG distribution at Mg sites, possibly due to increased structural disorder in Mg coordination environments or the presence of multiple distinct Mg sites with varying local structures, which cannot be resolved in the ^25^Mg spectra. These scenarios cannot be easily distinguished, as both would contribute to line broadening in ^25^Mg NMR.

**Figure 14:**
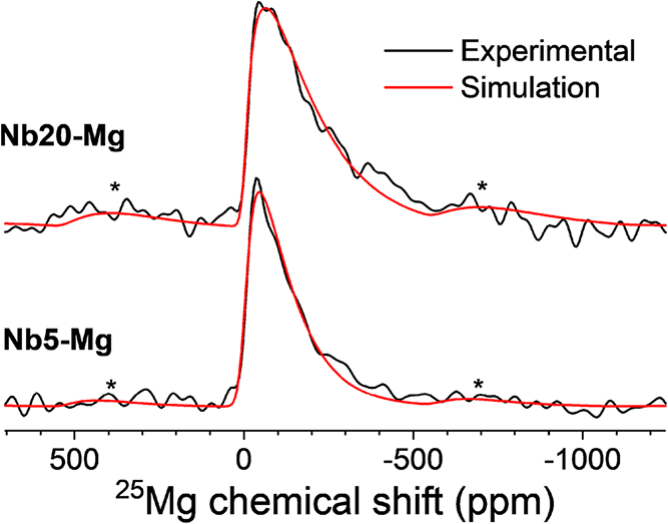
Experimental and simulated ^25^Mg spectra for Nb5–Mg and Nb20–Mg glass samples. The simulations were performed using an Extended Czjzek model, as described in the main text.

## 4 Conclusions

A new niobium-fluorophosphate glass matrix was synthesized via a conventional melt-quenching method. A comprehensive investigation was carried out by systematically varying both the Nb_2_O_5_ content (5, 10, 15, and 20%) and the type of alkaline earth metal (Mg^2+^, Ca^2+^, Sr^2+^, and Ba^2+^), introduced as their corresponding fluorides. As the Nb_2_O_5_ content increases, the glass structure evolves due to the progressive substitution of P–O–Nb bonds by Nb–O–Nb linkages, which have a higher degree of covalency and stronger chemical bonding. This structural transformation manifests through several effects: an increase in glass transition temperature (*T*_g_), an alteration of the phosphate units, and a red shift in the ultraviolet absorption edge, corresponding to a reduction in the optical bandgap. Refocused INADEQUATE ^31^P NMR spectroscopy was employed to monitor the evolution of P*^n^* phosphate units with an increasing niobium content. The addition of Nb_2_O_5_ leads to alteration of phosphate groups, promotes matrix interconnectivity through the formation of P_1Nb_^1^, and increases the population of P_2Nb_^2^ species.

The type of alkaline earth metal also significantly influenced the glass properties. *T*_g_ varied primarily as a function of Nb_2_O_5_ content; however, the crystallization temperatures (*T*_x_ and *T*_p_) and the thermal stability parameter (Δ*T*) were more strongly affected by the identity of the alkaline earth metal. Notably, Ca^2+^ containing glasses exhibited the highest *T*_x_, *T*_p_, and Δ*T*, with Δ*T* reaching nearly 400 °C for the Nb10–Ca composition, whereas Ba^2+^-containing glasses showed no detectable crystallization peaks.

Raman spectroscopy revealed that smaller alkaline earth cations promoted clustering of NbO_6_ octahedra, while larger cations induced the elongation of P–O and Nb–O bonds. Furthermore, ^19^F and ^25^Mg NMR measurements demonstrated a strong dependence of fluorine coordination on the ionic radius of the alkaline earth metal. F–Mg coordination was consistently intense across all Nb_2_O_5_ concentrations, while F–Ba interactions were not detected in any of the Nb*y*-Ba samples. These findings help explain the distinct thermal and optical behaviors observed, such as the variations in *T*_g_ and optical bandgap energies.

Although Mg^2+^ has a smaller ionic radius and a higher cationic field strength (*Z*/*r* = 2.78 Å^–1^), which strengthens the glass network connectivity and increases the glass transition temperature (*T*_g_), its strong affinity for fluoride ions reduces the availability of F^–^ to stabilize the phosphate chains. At the same time, Mg^2+^ promotes the clustering of NbO_6_ units, introducing structural heterogeneities that facilitate crystallization and, consequently, reduce the thermal stability against devitrification (Δ*T*).

Overall, this study provides valuable insights into how varying P/Nb ratios and the nature of the alkaline earth modifier affect the glass structure and its associated properties. The results support the rational design of fluorophosphoniobate glasses with tailored properties for advanced applications, particularly those requiring enhanced chemical and thermal stability.

## Supplementary Material



## Data Availability

All data related to this study are contained within the article and the Supporting Information. Data are available upon request.

## Notes

Photograph of all of the synthesized glass bulks (Figure S1); photograph of the glass bulks containing 5 mol % of Nb_2_O_5_ after exposure to environmental atmosphere (Figure S2); DSC thermograms related to all synthesized glasses (Figure S3); glass transition temperature of all glasses in the function of the alkaline earth metals’ ionic radii (Figure S4); UV–vis–NIR absorption spectra related to all glass bulks (Figure S5); Tauc plot used to calculate Urbach energy, direct and indirect optical bandgap energy values, exemplified for the sample Nb10–Mg (Figure S6); Raman spectra related to all glass bulks (Figure S7); ^31^P NMR spectra for all glasses (Figure S8); a comparison between ^31^P single-pulse excitation and INAD experiments for samples Nb5–Mg and Nb20–Mg and the deconvolution of ^31^P single-pulse excitation for Nb5–Mg spectrum (Figure S9); stacked Raman spectra for the Nb*y*-Ca sample set (Figure S10); ^23^Na NMR spectra for all glasses (Figure S11); ^23^Na NMR overlapping Nb*y*-Mg glasses (*y* = 5, 10, 15, and 20 mol % of Nb_2_O_5_) and Nb20-X glasses (X = Mg, Ca, Sr, and Ba) (Figure S12); ^23^Na­{^31^P} REDOR dephasing curves related to all glasses, containing the comparison with the crystalline Na_2_PO_3_F reference and parabolic fits for Δ*S*/*S*_0_ ≤ 0.25 (Figure S13); characteristic temperatures (*T*_g_, *T*_x_, *T*_p_, and Δ*T*) obtained from the DSC thermograms for all glasses (Table S1); calculated relative areas for each assigned phosphate group for sample Nb5–Mg (Table S2); and experimental second moments, *M*_2(Na‑P)exp_, obtained from the parabolic fit of the ^23^Na­{^31^P} REDOR experiments, along with the calculated number of phosphorus species, N_P_, for each glass (Table S3) (PDF)
