# Supplementary material for: Alkaline Earth Metal Fluorides (MgF2, CaF2, SrF2, BaF2) and Nb2O5 Effect on the Structural and Optical Properties of New Fluorophosphoniobate Glasses
Source: ACS Omega. 2025 Sep 26;10(45):54111–27. doi: 10.1021/acsomega.5c05892 (PMC12631399; doi:10.1021/acsomega.5c05892)
Supplement: Supplementary file 1 [file ao5c05892_si_001.pdf]

Supplementary Information

Alkaline earth metal fluorides (MgF_2, CaF_2, SrF_2, BaF_2) and Nb_2O_5 effect on the structural and optical properties of new fluorophosphoniobate glasses

Leandro Olivetti Estevam da Silva[1], *Lais Dantas Silva*[2], *Edgar Dutra Zanotto*[3], *Marcos de Oliveira Júnior*[4*], *Danilo Manzani*[1*]

L. O. E. S., Prof. D. M.
[1]São Carlos Institute of Chemistry (IQSC), University of São Paulo (USP),
São Carlos, SP, 13566-590, Brazil
*E-mail: dmanzani@usp.br

Prof. L.D.S.
[2] Department of Chemistry and Biology, State University of Maranhão (UEMA) Caxias, MA, 65604-380, Brazil.

Prof. Edgar Dutra Zanotto
[3]Center for Research, Technology, and Education in Vitreous Materials (CeRTEV), Department of Materials Engineering (DEMa), Federal University of São Carlos (UFSCar), São Carlos, SP, 13565-905, Brazil

Prof. M. O. J.
[4]São Carlos Institute of Physics (IFSC), University of São Paulo (USP),
São Carlos, SP, 13566-590, Brazil
*E-mail: mjunior@ifsc.usp.br

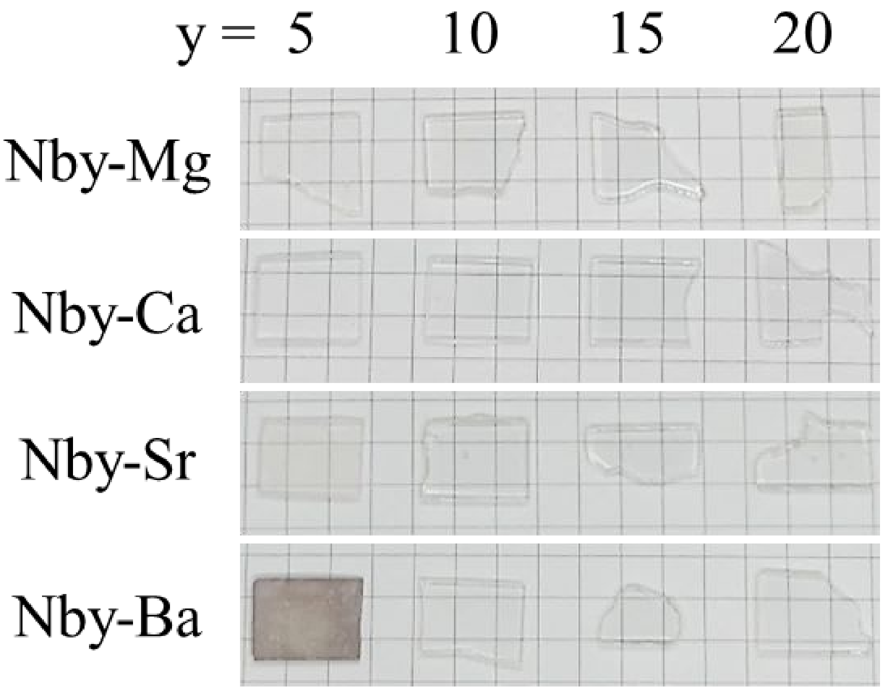

Figure S1. Photograph of Nby-X glass samples. Each line represents a set with a different alkaline earth metal contained in the samples, X = Mg, Ca, Sr and Ba, respectively; each column represents a concentration of Nb_2O_5 or "y" value, being 5, 10, 15 and 20 mol% of Nb_2O_5, respectively. The photograph was taken approximately 4 months after the synthesis.

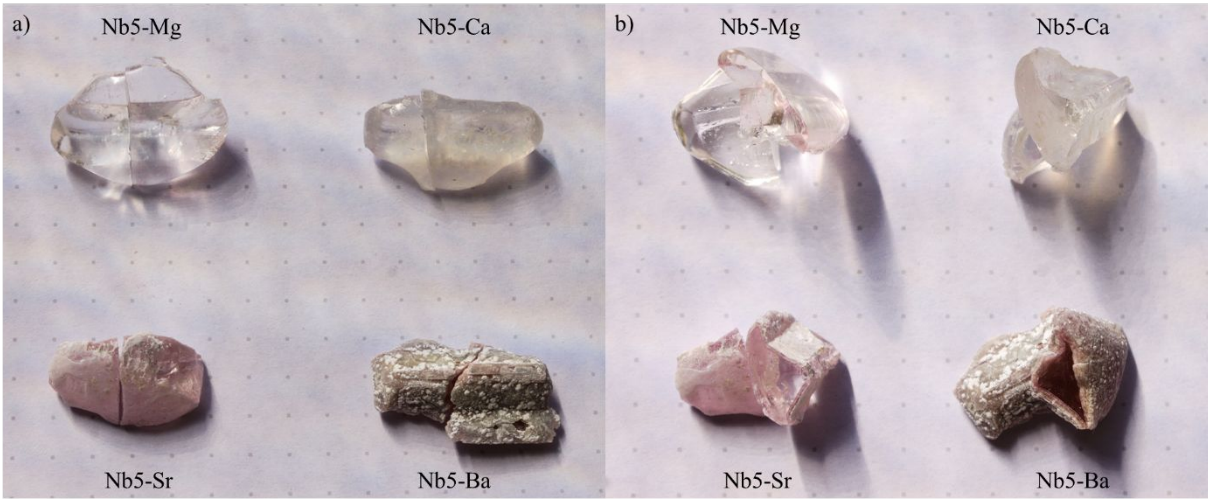

Figure S2. Photographs of Nb5-Mg, Nb5-Ca, Nb5-Sr and Nb5-Ba glass samples (as identified in the image) a) as bulk and b) cross-section of the bulk. The photograph was taken approximately 30 months after the synthesis, and the samples were recently snapped for the cross-section image.

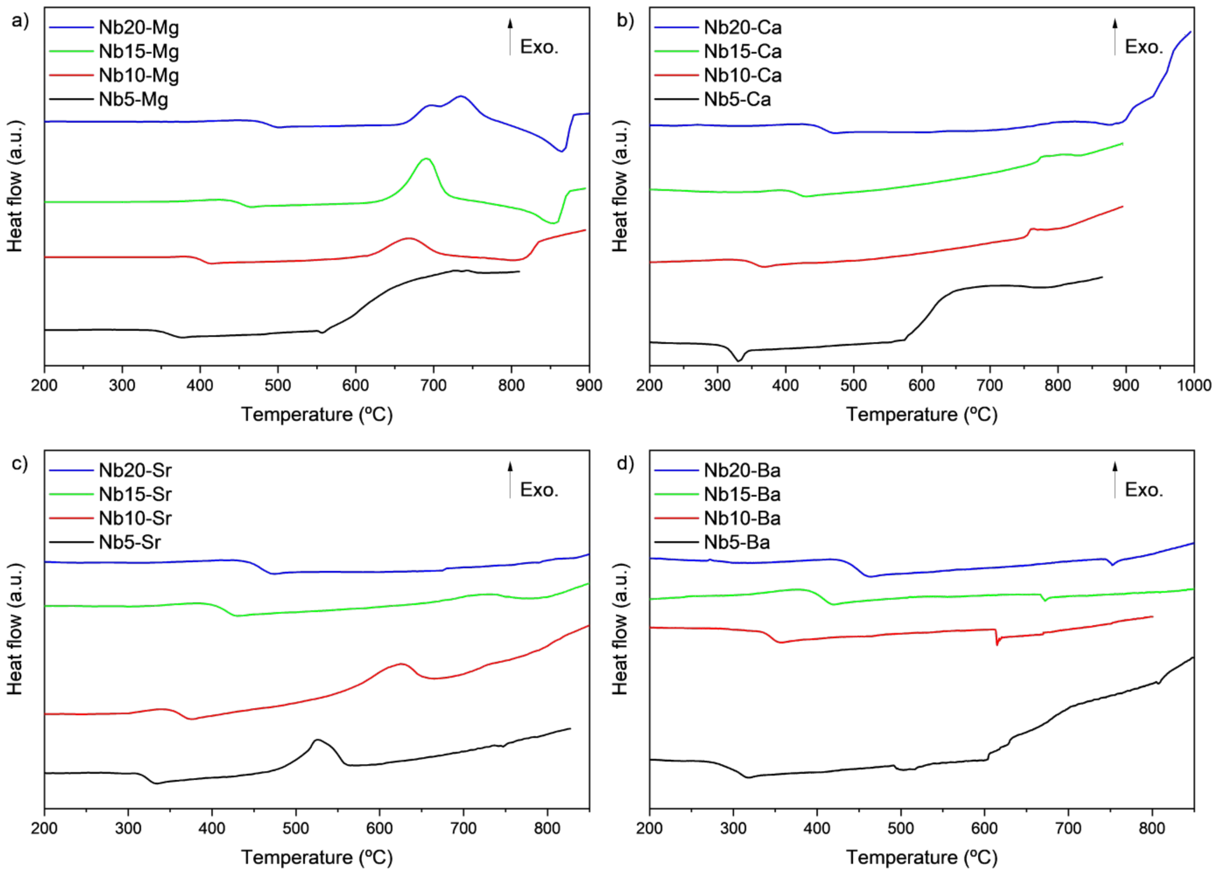

Figure S3. Thermogram obtained by DSC of all the Nby-X glass samples, with X = (a) Mg^{2+}, (b) Ca^{2+}, (c) Sr^{2+} and (d) Ba^{2+}.

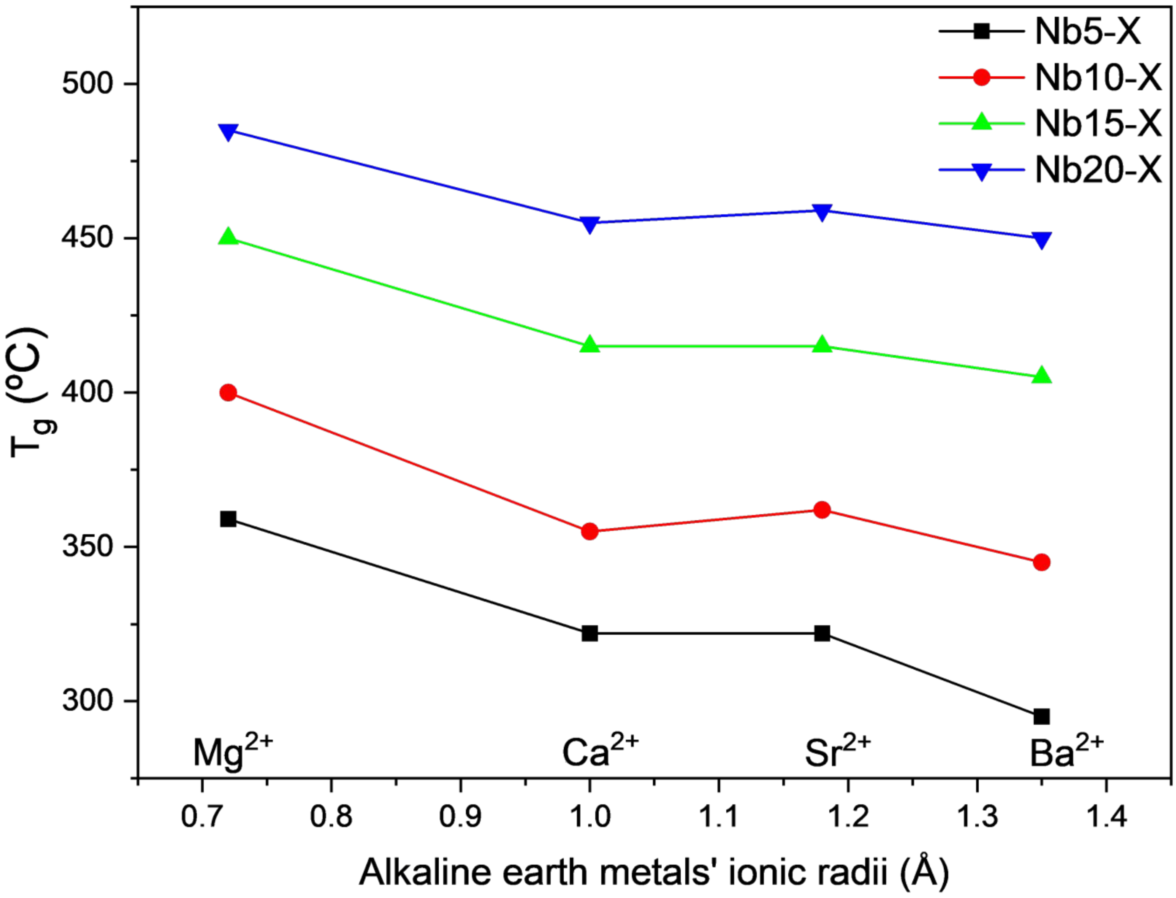

Figure S4. Glass transition temperatures (T_g) in function of the alkaline earth metals' ionic radii (Å). Ionic radii based on R. D. Shannon, considering the hexacoordinated state [38].

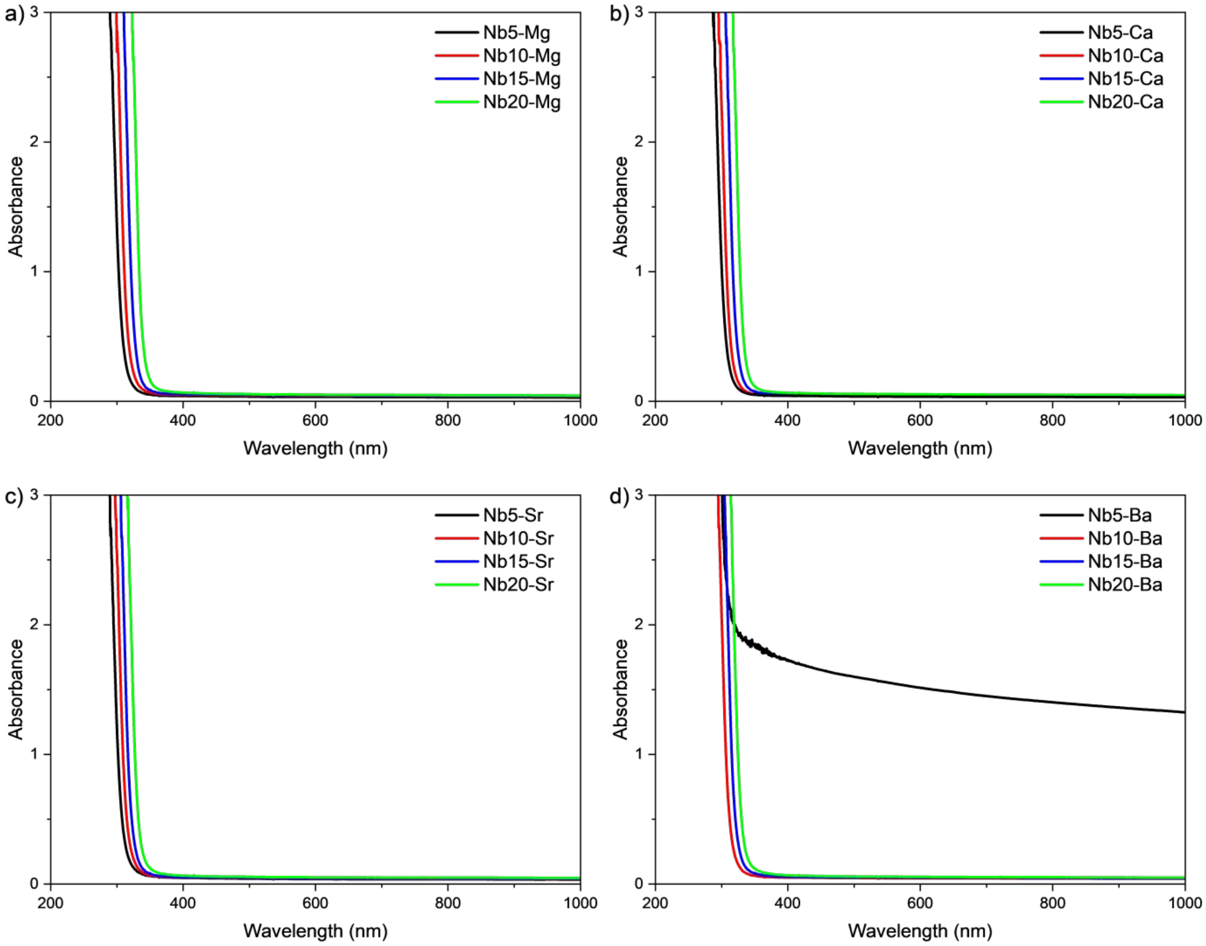

Figure S5. UV-Vis-NIR absorption spectra of the Nby-X glass samples, with y = 5, 10, 15 and 20 mol% and X = (a) Mg^{2+}, (b) Ca^{2+}, (c) Sr^{2+} and (d) Ba^{2+}.

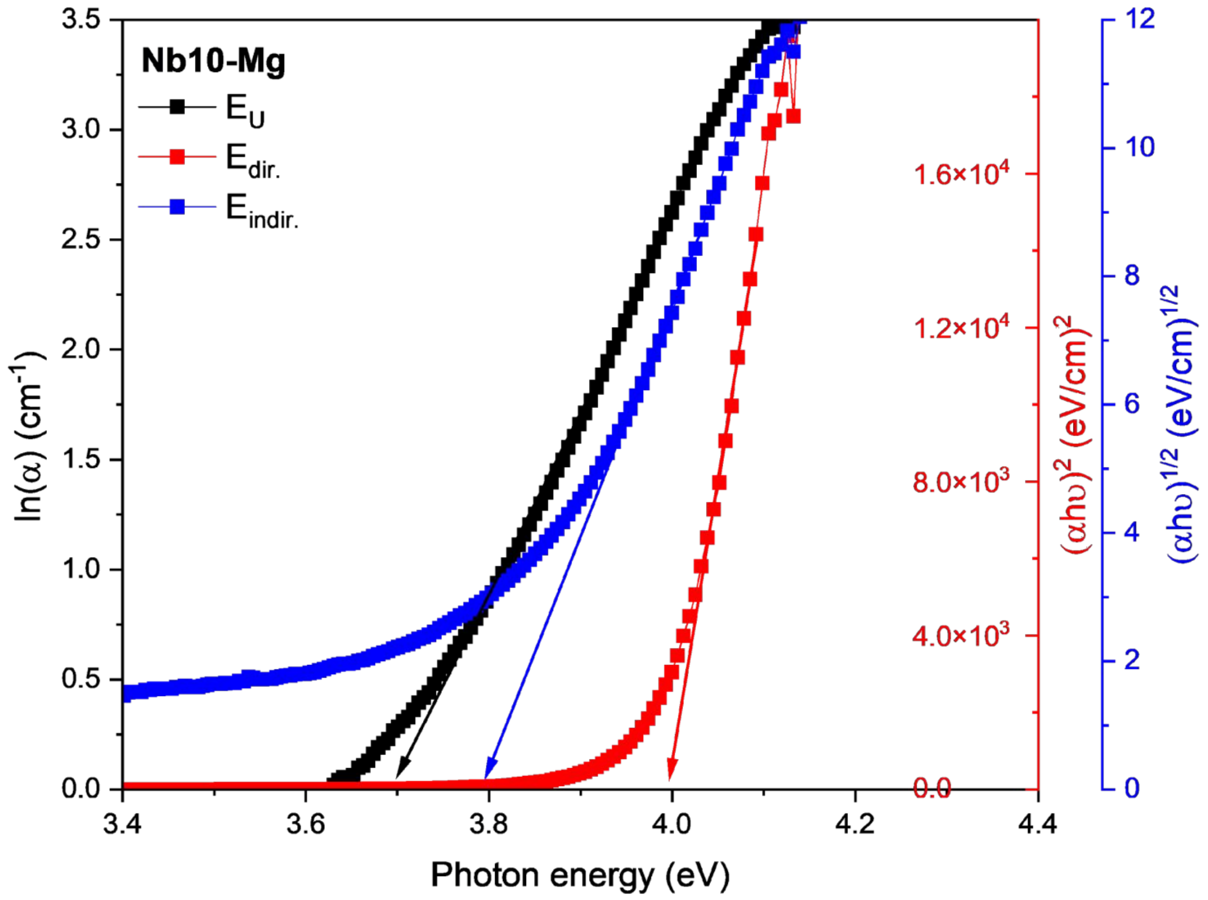

Figure S6. Tauc plot of Nb10-Mg glass sample, demonstrating how the Urbach energy, direct and indirect optical bandgap energy values were obtained for all Nby-X glass sample, plotting ln(α), (αhν)^2 and (αhν)$^{1/2}$, respectively, against the photon energy (eV).

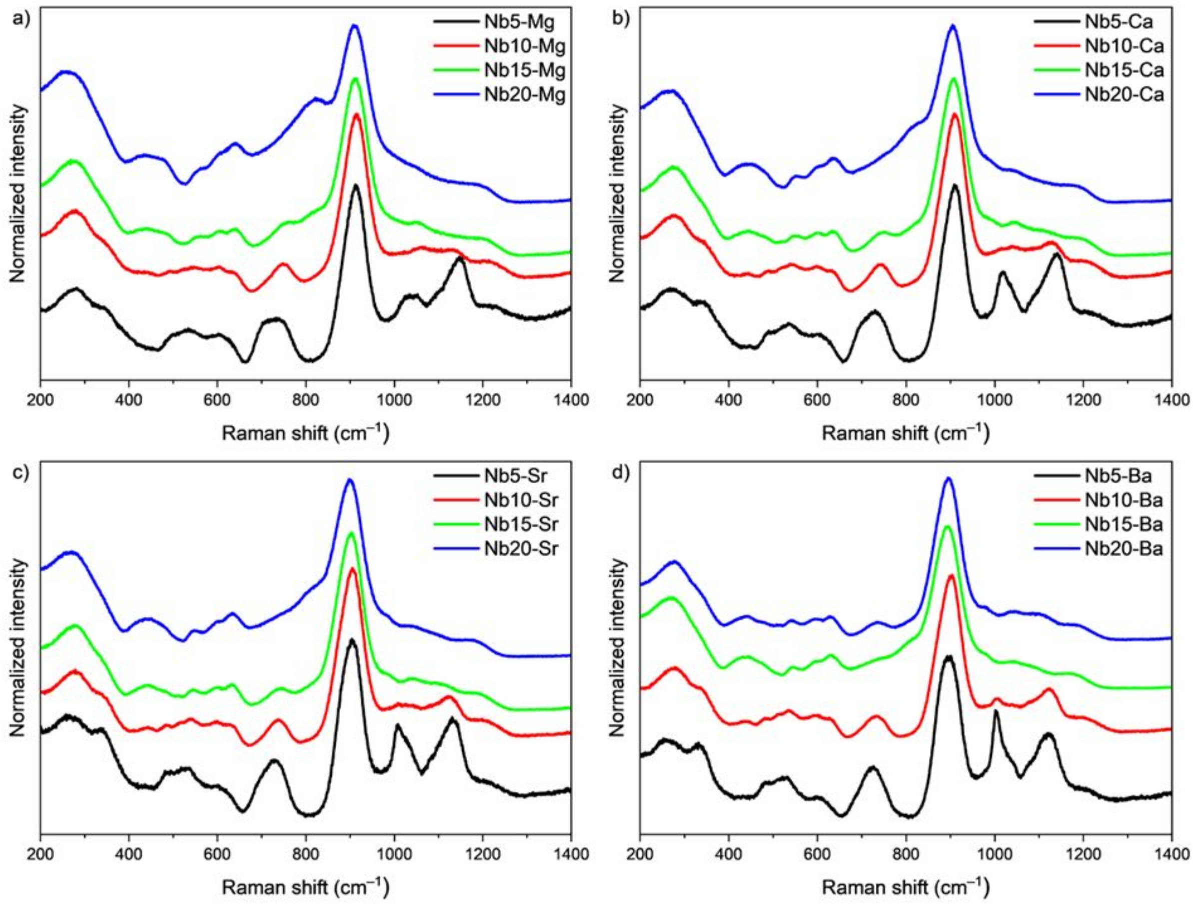

Figure S7. Raman spectra of Nby-X glass samples, with X = (a) Mg^{2+}, (b) Ca^{2+}, (c) Sr^{2+} and (d) Ba^{2+} and y = 5, 10, 15 and 20 mol% of Nb_2O_5.

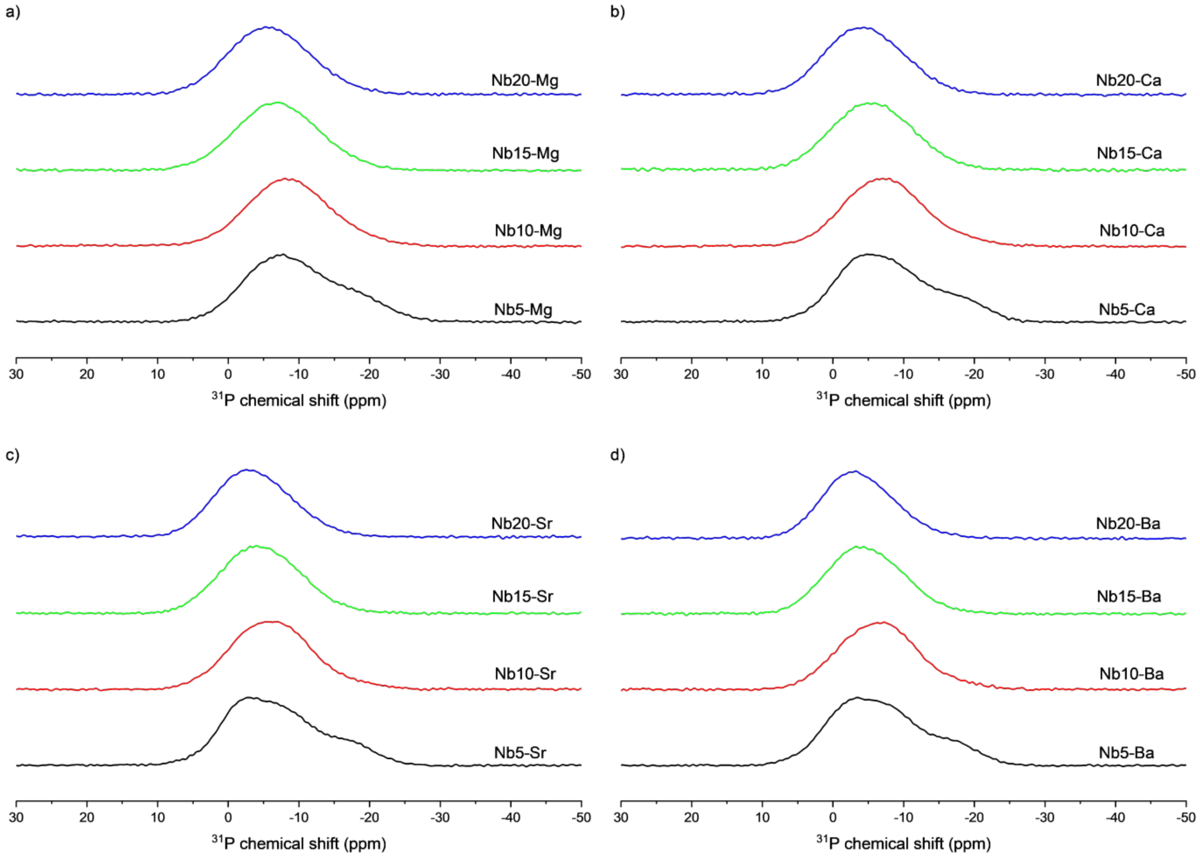

Figure S8. Set of nuclear magnetic resonance (NMR) spectra, monitoring the ^{31}P, of Nby-X samples, rotated at 15 kHz, with X = (a) Mg^{2+}, (b) Ca^{2+}, (c) Sr^{2+} and (d) Ba^{2+}.

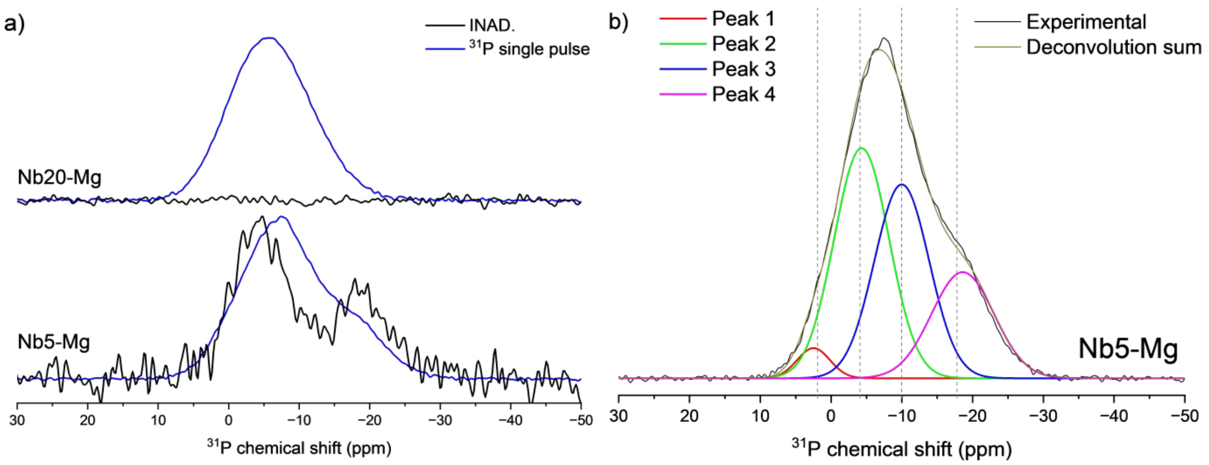

Figure S9. (a) Nuclear magnetic resonance spectrum of the Nby-Mg sample set (y = 5 and 20 mol% of Nb_2O_5), monitoring the ^{31}P, with experimental data from the single-pulse and refocused INADEQUATE spin-echo (INAD) experiments and (b) the deconvoluted single-pulse spectrum of Nb5-Mg glass, based on the gaussian fit from the INAD experiment.

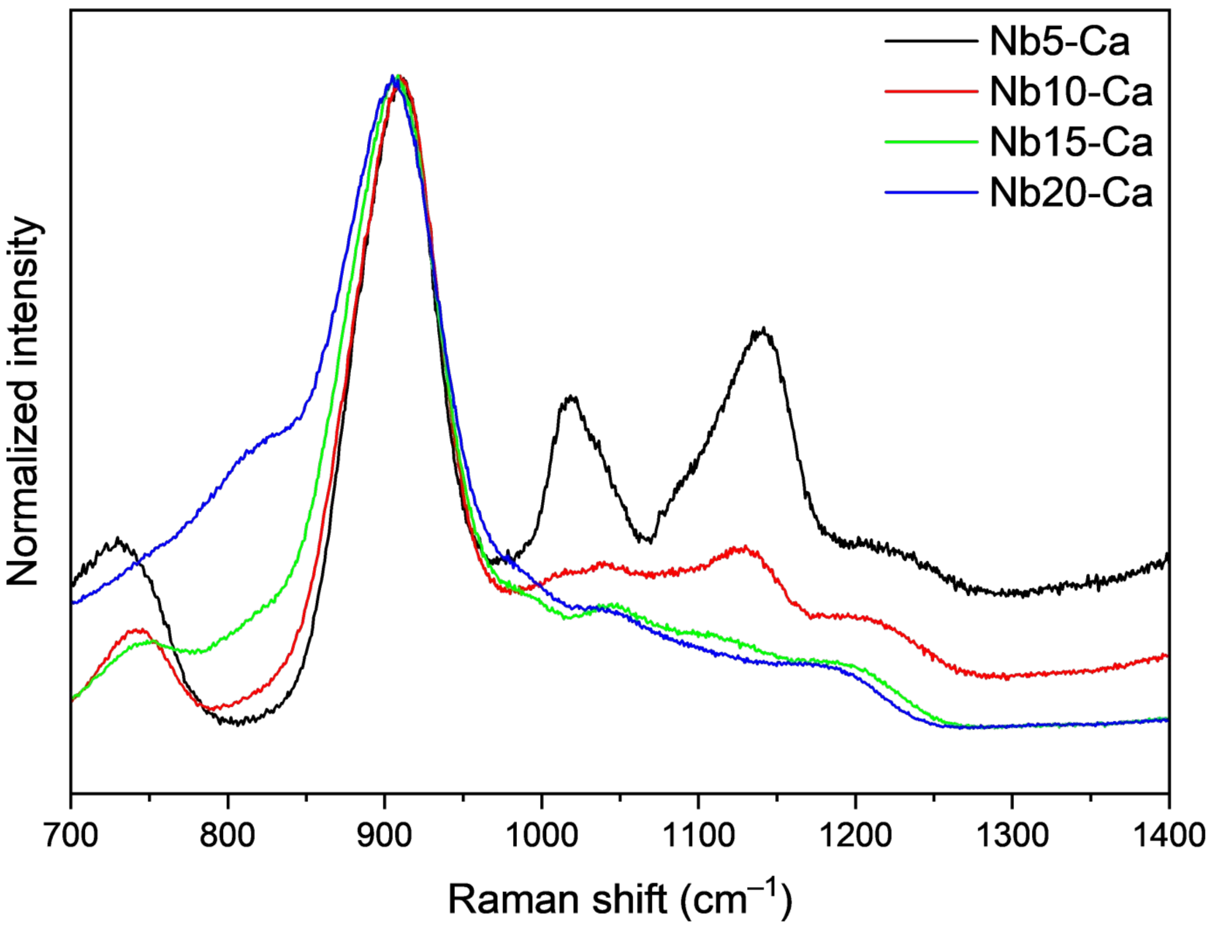

Figure S10. Stacked Raman spectra of all Nby-Ca glasses between 700 and 1400 cm^{-1}.

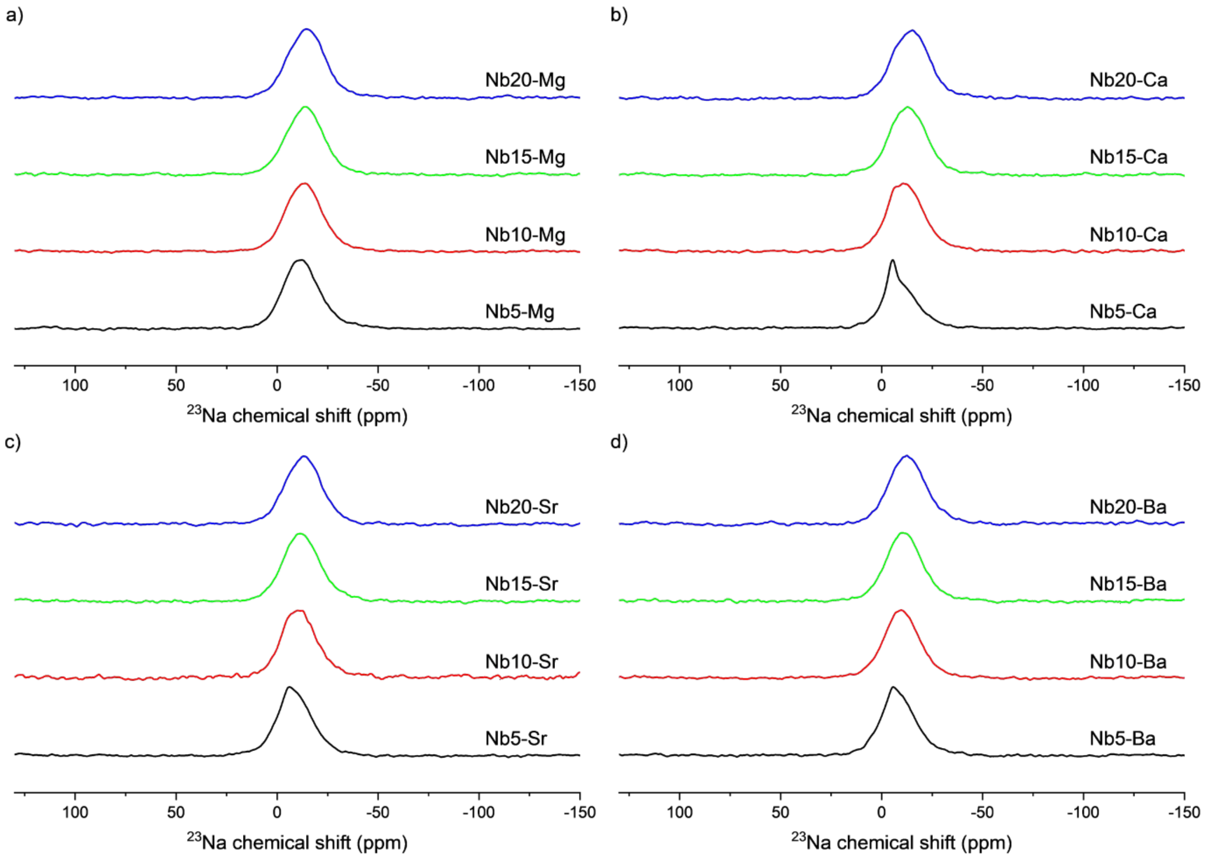

Figure S11. Set of nuclear magnetic resonance (NMR) spectra, monitoring the ^{23}Na, of Nby-X samples, rotated at 20 kHz, with X = (a) Mg^{2+}, (b) Ca^{2+}, (c) Sr^{2+} and (d) Ba^{2+}.

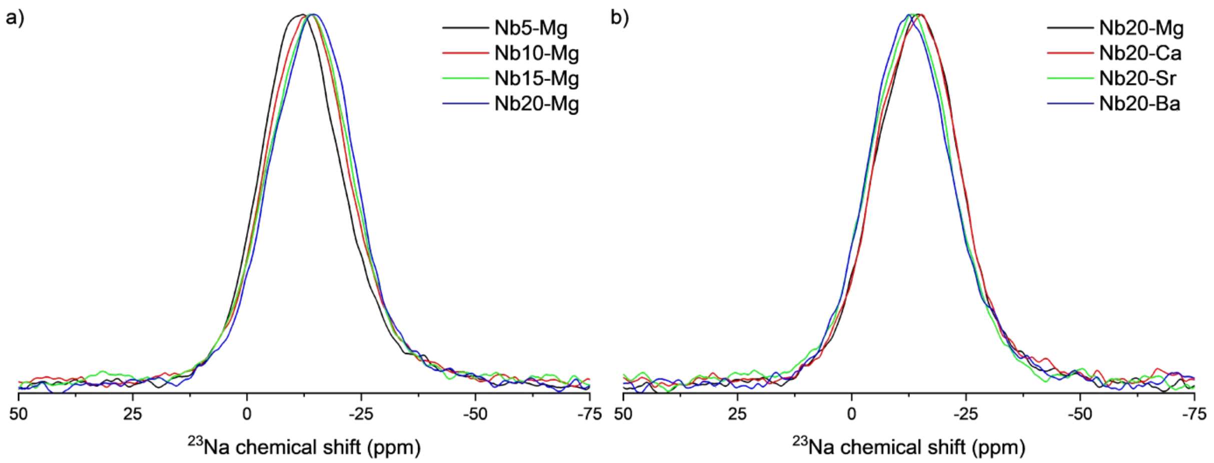

Figure S12. ^{23}Na NMR spectra representing the overlap of the a) Nby-Mg curves, y = 5, 10, 15 and 20 mol% of Nb_2O_5 and b) Nb20-X curves, X = Mg^{2+}, Ca^{2+}, Sr^{2+} and Ba^{2+}.

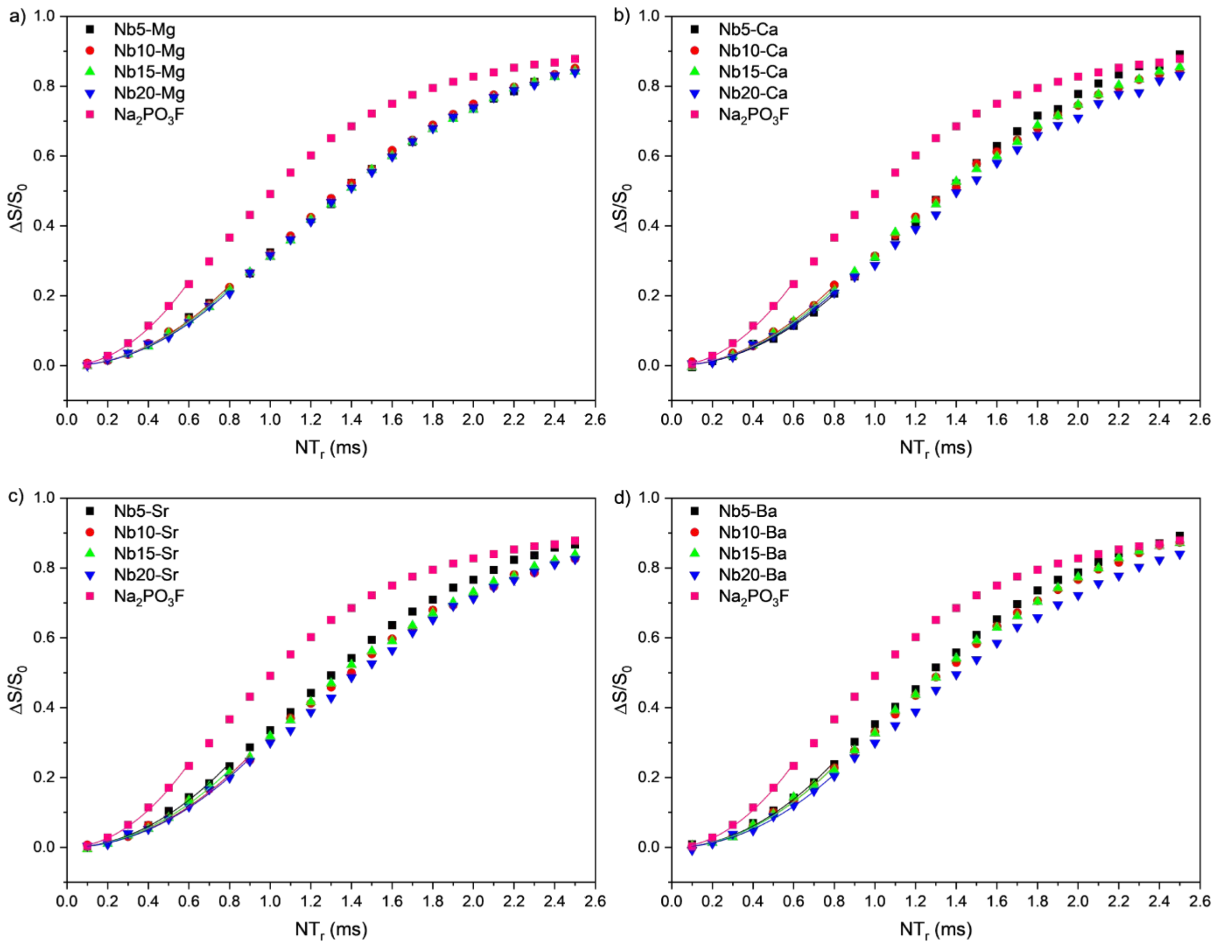

Figure S13. ^{23}Na{^{31}P} REDOR dephasing curves obtained for all Nby-X glasses, y = Mg^{2+} (a), Ca^{2+} (b), Sr^{2+} (c) and Ba^{2+} (d) and X = 5, 10, 15 and 20 mol% of Nb_2O_5, along with the reference measured for Na_2PO_3F crystal. Solid lines represent the parabolic fits to the data for $\Delta S/S_0 \leq 0.25$. The second moment M_2^{IS} values, related to the dipolar interaction between the quadrupolar observed nuclei (^{23}Na as observed nuclei, S = 3/2, and ^{31}P as non-observed nuclei, I = 1/2, in this case), were obtained by fitting the experimental REDOR data of $\Delta S/S_0$ (within the range of $0 \leq \Delta S/S_0 \leq 0.25$) with the expression $\frac{\Delta S}{S_0} = \frac{1}{I(I+1)\pi^2}(NT_r)^2 M_2^{IS}$ [63]. For obtaining a correction factor, f, between the $M_{2,exp}^{IS}$ and $M_{2,calc}^{IS}$ (experimental and calculated M_2^{IS}, respectively), this procedure was also used to measure a crystalline compound as reference, Na_2PO_3F. The value of M_2^{IS} can be calculated using the van Vleck expression [64], $M_2^{IS} = \frac{4}{15}\left(\frac{\mu_0}{4\pi}\right)^2 \gamma_I^2 \gamma_S^2 \hbar\, 2I(I+1)\sum_{i,j} \frac{1}{r_{IS}^6}$. The second moment values obtained from the parabolic fits are shown in Table S2. Also, by applying the f factor for all samples and comparing with the number of phosphorus atoms in the environment of the ^{23}Na nuclei, the number N_P of P atoms in the first coordination sphere of Na were obtained for all glasses. The values are also shown in Table S3.

Table S1. Characteristic temperatures obtained from DSC of Nby-X glass samples. T_g = glass transition temperature, T_x = offset crystallization temperature, T_P = onset crystallization temperature and ΔT = thermal stability against crystallization.

Samples	$T_g \pm 2$ °C	$T_x \pm 2$ °C	$T_p \pm 2$ °C	$\Delta T \pm 4$ °C
Nb5-Mg	359	-	-	-
Nb10-Mg	400	609	670	209
Nb15-Mg	450	645	690	195
Nb20-Mg	485	660	695	175
Nb5-Ca	322	580	647	258
Nb10-Ca	355	752	760	397
Nb15-Ca	415	767	775	352
Nb20-Ca	455	718	825	363
Nb5-Sr	322	486	525	164
Nb10-Sr	362	559	624	197
Nb15-Sr	415	675	726	260
Nb20-Sr	459	-	-	-
Nb5-Ba	295	-	-	-
Nb10-Ba	345	-	-	-
Nb15-Ba	405	-	-	-
Nb20-Ba	450	-	-	-

Table S2. Assigned phosphate groups and relative areas related to each peak (in %) from the deconvolutions of Nb5-Mg sample, in Figure S9 (b).

Assigned phosphate groups	Chemical shift (ppm)	Relative area (%)
$P^0 + P^1_{1Nb}$	2.5	3
P^1	–4.3	41
P^2_{2Nb}	–9.9	34
$P^2_{1Nb} + P^2_{0Nb}$	–18.6	21

Table S3. Experimental second moments ($M_{2\ (Na–P)\ exp.}/10^6\ rad^2\ s^{-2}$) obtained for all samples and the respective number of phosphorus (N_P) calculated for each one, based on the calculated second moment for the reference compound, Na_2PO_3F, as $4.95\times10^6\ rad^2\ s^{-2}$, and the f factor calculated as ~1.

**Sample**	$\mathbf{M_{2\ (Na–P)\ exp.}}$ ± 0.2	$\mathbf{N_P}$ ± 0.2
$\mathbf{Na_2PO_3F}$	4.9	6.0
**Nb5-Mg**	2.7	3.2
**Nb10-Mg**	2.7	3.2
**Nb15-Mg**	2.6	3.1
**Nb20-Mg**	2.5	3.0
**Nb5-Ca**	2.4	2.9
**Nb10-Ca**	2.7	3.2
**Nb15-Ca**	2.5	3.1
**Nb20-Ca**	2.4	3.0
**Nb5-Sr**	2.8	3.4
**Nb10-Sr**	2.4	2.9
**Nb15-Sr**	2.6	3.1
**Nb20-Sr**	2.3	2.8
**Nb5-Ba**	2.8	3.4
**Nb10-Ba**	2.7	3.3
**Nb15-Ba**	2.7	3.2
**Nb20-Ba**	2.4	2.9
